# A mutation in THREONINE SYNTHASE 1 uncouples proliferation and transition domains of the root apical meristem: experimental evidence and *in silico* proposed mechanism

**DOI:** 10.1242/dev.200899

**Published:** 2022-11-09

**Authors:** Monica L. García-Gómez, Blanca J. Reyes-Hernández, Debee P. Sahoo, Selene Napsucialy-Mendivil, Aranza X. Quintana-Armas, José A. Pedroza-García, Svetlana Shishkova, Héctor H. Torres-Martínez, Mario A. Pacheco-Escobedo, Joseph G. Dubrovsky

**Affiliations:** ^1^ Departamento de Biología Molecular de Plantas, Instituto de Biotecnología, Universidad Nacional Autónoma de México (UNAM), Av. Universidad, 2001, Cuernavaca 62250, Mexico; ^2^ Facultad de Ciencias de la Salud, Universidad Tecnológica de México – UNITEC MÉXICO – Campus Atizapán, Av. Calacoaya 7, Atizapán de Zaragoza, Estado de México, 52970, Mexico

**Keywords:** Root apical meristem, Stem cells, Transit-amplifying cells, Threonine metabolism, Meristem maintenance, Meristem exhaustion, *Arabidopsis thaliana*

## Abstract

A continuum from stem to transit-amplifying to a differentiated cell state is a common theme in multicellular organisms. In the plant root apical meristem (RAM), transit-amplifying cells are organized into two domains: cells from the proliferation domain (PD) are displaced to the transition domain (TD), suggesting that both domains are necessarily coupled. Here, we show that in the *Arabidopsis thaliana mto2-2* mutant, in which threonine (Thr) synthesis is affected, the RAM lacks the PD. Through a combination of cell length profile analysis, mathematical modeling and molecular markers, we establish that the PD and TD can be uncoupled. Remarkably, although the RAM of *mto2-2* is represented solely by the TD, the known factors of RAM maintenance and auxin signaling are expressed in the mutant. Mathematical modeling predicts that the stem cell niche depends on Thr metabolism and that, when disturbed, the normal continuum of cell states becomes aborted.

## Teaser

**Highlighted Article:** Experiments and mathematical modeling suggest that to maintain the functional root apical meristem*,* its stem cell niche depends on threonine metabolism and modeling correctly predicts root growth pattern.

## INTRODUCTION

In plants and animals, continuous tissue replenishment relies on the long-term maintenance of undifferentiated cells with the capacity to self-renew and produce a progeny of specialized cell types. Longitudinal plant root growth depends on cell production in the root apical meristem (RAM), the function of which in turn depends on the activity of stem (initial) cells located at the periphery or directly surrounding (depending on species) a population of rarely dividing cells called the quiescent center (QC) (Clowes, 1956; Dolan et al., 1993; Dubrovsky and Ivanov, 2021). Both the QC and stem cells make up a stem cell niche (SCN) (Sabatini et al., 2003; Wildwater et al., 2005). Stem cell derivatives enter the two domains of transit-amplifying cells of the RAM successively: the proliferation domain (PD), in which cells actively proliferate but grow slowly, approximately doubling their size during a cell cycle, and the transition domain (TD), in which, before rapid elongation, cells decrease their proliferation activity and transit to endoreduplication, but continue to grow at the same relative growth rate (Dubrovsky and Ivanov, 2021; Hayashi et al., 2013; Ivanov and Dubrovsky, 2013). Thus, the RAM is organized into two domains, the PD and TD, and it appears that these domains are inevitably coupled, as the cells in the TD are those that were formed in and displaced from the PD (Ivanov and Dubrovsky, 2013). Subsequently, the cells start rapid anisotropic growth within the elongation zone (EZ), and fully elongated cells acquire the final differentiated state in the differentiation zone.

Over recent decades, several regulatory pathways involved in RAM maintenance have been identified. Classical players in these pathways are the transcription co-factors from the GRAS family, SHORTROOT (SHR) and SCARECROW (SCR), and the AP2-transcription factors PLETHORA (PLT). The single *shr* or *scr* and the double *plt1 plt2* loss-of-function mutants are not capable of RAM maintenance and show RAM exhaustion and determinate root growth (Aida et al., 2004; Galinha et al., 2007; Helariutta et al., 2000; Nakajima et al., 2001; Sabatini et al., 2003). The coordinated role of some of these transcription factors with hormones has been unraveled with the aid of mathematical models (Cruz-Ramírez et al., 2012; Salvi et al., 2020b), and new experimental data show that they act in concert with peptides and other factors to maintain the quiescence of the QC and RAM activity (Bertolotti et al., 2021; Pardal and Heidstra, 2021; Santuari et al., 2016; Shimotohno et al., 2018; Strotmann and Stahl, 2021). Among the main factors involved in maintaining the quiescence of the QC cells are WUSCHEL RELATED HOMEOBOX 5 (WOX5) and BRASSINOSTEROIDS AT VASCULAR AND ORGANIZING CENTER (BRAVO) transcription factors (Betegón-Putze et al., 2021; Forzani et al., 2014; Sarkar et al., 2007; Vilarrasa-Blasi et al., 2014). Recently, through forward genetic screening, we identified two mutants with a determinate root growth phenotype. These mutants are affected in *FOLYLPOLYGLUTAMATE SYNTHETASE 1* (*FPGS1*; Reyes-Hernández et al., 2014) and *METHIONINE OVERACCUMULATOR 2* (*MTO2*; Reyes-Hernández et al., 2019), pointing to a crucial role of the metabolism in the maintenance of the quiescence of the QC and the RAM function. Loss-of-function mutants in both genes develop an extremely short primary root in comparison with wild type (Wt) plants, which is the result of the gradual consumption of its RAM leading to its exhaustion (Hernández-Barrera et al., 2011; Reyes-Hernández et al., 2019, 2014).

The *MTO2* gene encodes THREONINE SYNTHASE 1 (TS1), revealing a link between threonine (Thr) metabolism and the maintenance of the indeterminate growth program in the root; however, the mechanism remains unknown. Roots of the *mto2-2* mutant (possessing a point mutation in the *MTO2* open reading frame; previously called *mko1*) display a decreased activity of the G2-M marker *CycB1;1_DB_::GFP* (Hernández-Barrera et al., 2011), indicating a crucial role of Thr in cell proliferation in the RAM that may explain its determinate growth phenotype. Curiously, free Thr levels in the *mto2-2* root system are actually 59% higher than in Wt but, when exogenous Thr is added, the RAM of the primary root reestablishes its activity (Reyes-Hernández et al., 2019)*,* suggesting a compartmentalization of Thr along the root. Still, the distribution of free Thr in the RAM remains to be determined, and contemporary methods do not permit such an analysis. This knowledge could be crucial to establish a Thr-dependent mechanism of RAM maintenance and indeterminate growth. Previous data (Hernández-Barrera et al., 2011) and data presented in this paper show that known markers of SCN activity are expressed in the root apex until advanced stages of meristem consumption. On the other hand, the diminished cell proliferation activity results in RAM cortical cells being longer in the mutant than in the Wt (Hernández-Barrera et al., 2011). Despite the fact that the RAM domains could be altered in the mutant, the existence of the PD and TD domains have not been previously explored; here, we address the hypothesis that the *mto2-2* mutant lacks the PD.

The nature of the TD is debatable and sometimes it is considered to be a separate zone, neither belonging to the RAM nor to the EZ (Ivanov and Dubrovsky, 2013; Salvi et al., 2020a; Yamoune et al., 2021). In this study, we used experimental and mathematical modeling approaches to uncover the mechanism linking Thr metabolism with RAM maintenance. The experimental and computational results indicated that in the *mto2-2* mutant only the TD is present, and thus *MTO2* plays a key role in coupling the PD and TD of the RAM*.* Through mathematical modeling we leveraged previously published transcriptomic data of the enzymes involved in Thr metabolism to predict and subsequently evaluate Thr distribution along the root growth zones in Wt and the *mto2-2* mutant. The *in silico* root model presented here predicts an essential role of Thr in defining the organization of the RAM domains and reveals the SCN as the region most affected in Thr metabolism in *mto2-2*. As stem cells are the ultimate source of new cells in plant organs, and as such they are essential for RAM activity and indeterminate organ growth, our results suggest that a malfunction of the SCN in the *mto2-2* mutant caused by Thr depletion leads to lack of stem cell activity and the gradual consumption of the RAM.

## RESULTS

### A mutation in *MTO2* affects the RAM cell length profile

**Fig. 1.**
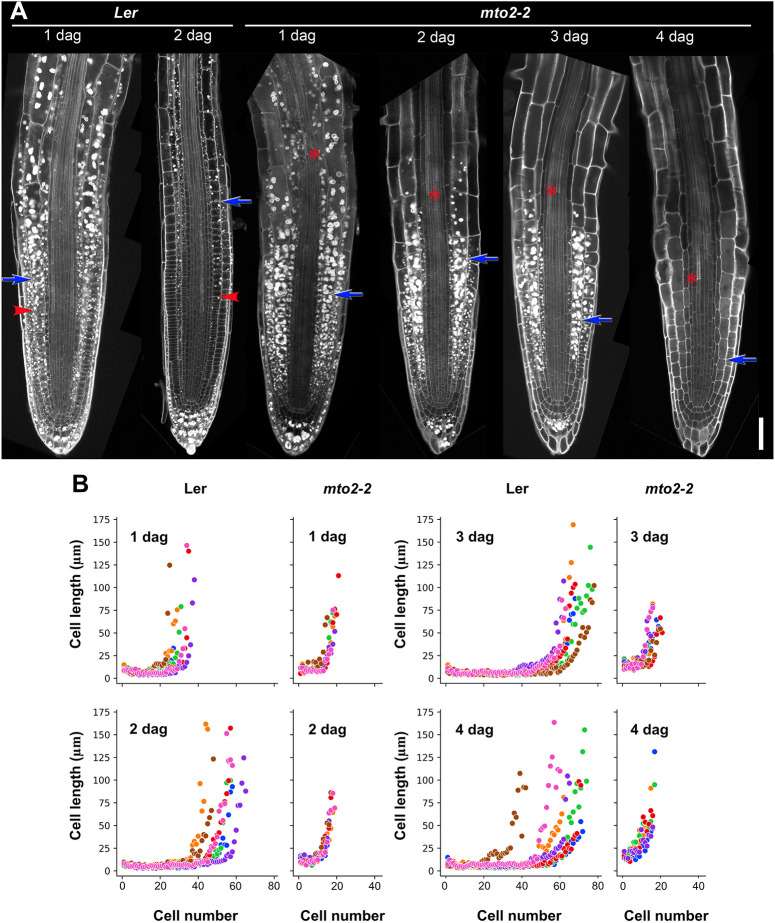
**Differences in the establishment of the RAM domains in Wt and *mto2-2* mutant roots.** (A) Representative roots of Wt and *mto2-2* seedlings stained with Pseudo-Schiff reagent; all images are at the same magnification. Scale bar: 50 µm. Arrows and arrowheads indicate the RAM boundary and the boundary between the PD and TD, respectively. Asterisks indicate a position of the most distal differentiated xylem. (B) Cell length profile in roots of Wt and *mto2-2* seedlings in one independent experiment (*n*=7). Cell lengths for an individual root are marked with the same color. As the transition to elongation in different roots was at a different distance from the QC, no average values were estimated. Cortex cell length was measured. See also Fig. S1.

Previously we isolated the *mko1* (‘short root’ in Mayan) mutant, the RAM of which becomes exhausted (developmentally lost or consumed) (Hernández-Barrera et al., 2011). We have identified that this phenotype is caused by a point mutation in the *MTO2* open reading frame that encodes TS1 and renamed the mutant as *mto2-2* (Reyes-Hernández et al., 2019). In the *mto2-2* mutant, a normal embryonic root (radicle) is established during embryogenesis (Hernández-Barrera et al., 2011). At 0 days after germination (DAG), when the radicle just protrudes the testa (∼48 h after beginning of seed imbibition), no signs of cell division were detected and the average length of the distal ten cells in the radicle of the mutant and Ler Wt (hereafter Wt) was similar (Fig. S1A), confirming that the mature embryo in the mutant is similar to that of Wt. We chose to measure ten distal cells because our analysis showed that this number is close to the RAM length in the *mto2-2* mutant before the RAM exhaustion (see below and Table S1). Consistent with previous reports (Salvi et al., 2020b), in 1 DAG Wt seedlings the RAM started its activity, and short meristematic cells could be appreciated in the RAM (Fig. 1A). At this age, in *mto2-2* seedlings, the average cortex cell length of ten distal RAM cells was 1.6-fold greater than those in Wt (*P*<0.001, Student's *t*-test; Fig. S1A), suggesting possible differences in the RAM establishment. In the *mto2-2* mutant, a complete RAM exhaustion is detected in seedlings starting from 5 DAG (Reyes-Hernández et al., 2019). To investigate the differences in the establishment of the RAM domains, we built cell length profiles using 1-4 DAG plants, before the RAM exhaustion was detected in *mto2-2*, and found that in 1 DAG Wt RAM the PD and TD of the RAM were already established. In Wt plants starting from 2 DAG, starch reserves were already consumed, whereas in the *mto2-2* mutant they were present until 3 DAG (Fig. 1A), suggesting a delay in starch hydrolysis. In the mutant RAM at 1-4 DAG, no clear PD could be detected, and both RAM length and the distance to differentiated protoxylem were gradually decreased (Fig. 1A). Cell length measurements showed that the *mto2-2* RAM cells were of a greater length than those in the Wt (Fig. 1B; Fig. S1B-D). This analysis suggested that only one domain is present in the mutant RAM before its exhaustion. Because of the RAM cell length differences, we hypothesized that only TD was present in the RAM of the *mto2-2*.

### *In silico* root modeling predicts that the *mto2-2* mutant lacks the RAM PD

**Fig. 2.**
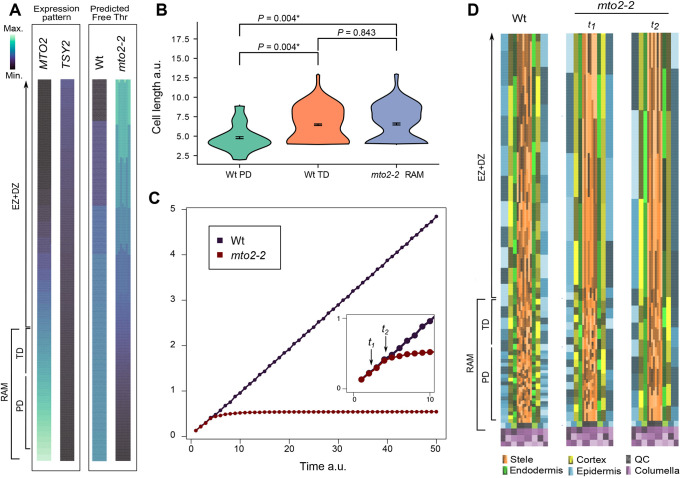
**Mathematical model of free Thr distribution in the root tip and prediction of root cell length profile in wild type and the *mto2-2* mutant.** (A) Expression patterns of *MTO2* and *TSY2* along the root, as defined by linear functions based on expression data (Wendrich et al., 2017; see Materials and Methods), and prediction of the free Thr distribution in Wt and the *mto2-2* roots. (B) Cell length distribution in the *in silico* model roots at a time between *t_1_* and *t_2_* (shown in C) when the RAM is still present. The length of RAM cells of *mto2-2* (241 cells) is similar to that of TD cells in Wt (90 cells), but not to that of PD cells (187 cells); *P* was calculated with non-parametric Mann–Whitney *U*-test. (C) Root growth curves of Wt and *mto2-2 in silico* roots. The inset showing *t_1_* and *t_2_* indicates the period where RAM cell length in *mto2-2* is statistically indistinguishable from that of Wt TD cells. (D) Cell length distribution in the *in silico* root, specifically within the RAM PD and TD and the elongation and differentiation zones (EZ+DZ). The simulations are shown for Wt at time *t_1_* and for the mutant at times *t_1_* and *t_2_* as indicated in C. Note that, although the root cap is depicted here for simplicity, in the *mto2-2* plants at time *t_2_* only the remains of the root cap are present, or it is absent. To appreciate cell length distributions along the *in silico* root, each subsequent cell in a cell file is depicted with a different tonality. Note that indications of the PD and TD of the RAM refer only to Wt and the *mto2-2* at time *t_1_*; in the *mto2-2* mutant at time *t_2_*, cell length profile reproduces absence of PD cells similar to real data shown in Fig. 1B. See also Movie 1.

To gain insight into the mechanism by which *MTO2* and free Thr impact RAM organization and root growth, we developed a mathematical model based on the two genes encoding for enzymes involved in Thr synthesis in A. *thaliana*: *MTO2* (*AT4G29840*; Bartlem et al., 2000) and *THREONINE SYNTHASE 2* (*TSY2*; *AT1G72810*; Curien et al., 2009; Morneau et al., 2013), which display opposite expression gradients in the root (Fig. 2A; see Materials and Methods). Currently, because of a lack of molecular markers or biosensors for free Thr detection, it is not feasible to measure its content at a cellular resolution. Here, using the power of modeling, we have attempted to predict the distribution pattern of free Thr in the SCN, the rest of the RAM, and the EZ and differentiation zone (DZ) using the expression patterns of the Thr synthase enzymes. The expression patterns implemented in our simulations are supported by data from public transcriptomic datasets (Brady et al., 2007; Wendrich et al., 2017) including recent single-cell transcriptomics of the root apex (Denyer et al., 2019; Zhang et al., 2019). Importantly, for *MTO2* and *TSY2* these open resources show good agreement among them, and the reported expression patterns were implemented in the model. In addition, we also performed a quantitative analysis of GPF intensity along the *pMTO2:MTO2-GFP* root tip, as a proxy for MTO2 protein distribution, and qRT-PCR analysis of *MTO2* and *TSY2* transcript abundance, and found, as expected, a clear *MTO2* gradient in Wt with a drastic decrease towards the DZ and an opposite gradient for *TSY2* (Fig. S2).

To build a minimal mathematical ordinary differential equation (ODE) model of free Thr distribution, we considered the role of MTO2 and TSY2 as well as the previously reported differences in the activity of these enzymes (Curien et al., 2009). This ODE model was then included in each cell of an *in silico* root with the following developmental zones: a RAM with an SCN comprising the QC and the provascular stem (initial) cells (other stem cells were not considered for simplicity), a PD, TD, and finally EZ and DZ (Fig. 2D). In this *in silico* root we superimposed the opposite transcript gradients of *MTO2* and *TSY2*, which led to the prediction that free Thr is distributed in a graded pattern with very high levels in the RAM, probably the highest in the SCN region, followed by a gradual decrease towards the DZ (Fig. 2A). This robust pattern emerges as a consequence of the expression pattern of the genes coding for enzymes directly involved in Thr biosynthesis, *MTO2* and *TSY2*.

Next, in the ODE describing free Thr biosynthesis we removed the contribution of MTO2 and included the putative compensatory activity of TSY2 (Curien et al., 2009) in order to simulate the *mto2-2* mutant root. Importantly, *mto2-2* is not a null mutant, but in the model we evaluate the complete loss of *MTO2* function, predicting a deficiency of free Thr in the RAM (Fig. 2A). The root phenotype of *mto2-2* strongly suggests a critical role of free Thr for cell proliferation and RAM maintenance (Hernández-Barrera et al., 2011). With an intention to reproduce this phenotype *in silico*, we linked the predicted free Thr distribution with the developmental decisions along the *in silico* root, specifically to the capacity of cells to divide in the RAM. Therefore, the model puts forward the hypothesis that a minimal level of free Thr must be available for a cell to divide within the RAM PD.

The Wt simulation recovered an indeterminate root growth due to the predicted enrichment of free Thr in the RAM (Fig. 2A,D), supporting high proliferation activity of the cells in the PD. In contrast, the *mto2-2* simulation resulted in an *in silico* root that grew only for a short period of time before reaching a plateau (Fig. 2C,D; Movie 1), consistent with the determinate root growth pattern established experimentally for *mto2-2* (Hernández-Barrera et al., 2011; Reyes-Hernández et al., 2019). The very limited growth of *mto2-2* was driven by cell elongation of the existing cells defined in the initial condition, and by very few new cells produced by limited cell divisions. During the brief period of growth of the *mto2-2 in silico* root, we noticed that the lack of cell divisions in the *mto2-2* simulations resulted in longer RAM cells in comparison with the Wt simulations (Fig. 2D). To describe in more detail the apparent differences between the Wt and the mutant, we statistically compared the cell length distributions of both RAMs. We found that, at a time point when the *in silico* RAM was still present in the *mto2-2* mutant, the length of its cells was statistically different from that of the Wt PD cells, whereas it was statistically indistinguishable from that of the Wt TD cells (Fig. 2B,D). This model result strongly suggests that, during the initial growth period, the RAM cells in *mto2-2* roots behave as TD cells and that the PD is not present. This severe compromise in RAM organization is deduced *in silico* exclusively from the predicted deficiency of free Thr in the root apex leading to insufficient cell proliferation in the *mto2-2* RAM. Consequently, as most RAM cells continue to grow slowly but do not divide, they behave as cells of the TD of Wt roots. These simulations suggest that TSY2 activity, particularly in the DZ, explains the higher free Thr content in whole roots of the *mto2-2* mutant compared with the Wt (Fig. S2,S3).

Overall, the results of the model support the crucial role of free Thr compartmentalization to maintain the activity of the RAM and predict that only one domain is present in the RAM of *mto2-2* roots. This is consistent with the difficulty in defining the domains in the *mto2-2* RAM based on the cell length profile (Fig. 1B) or arbitrarily (Fig. 1A). As the model predicts that only the TD domain of the RAM is present in the *mto2-2* mutant, we next addressed estimation of the RAM domains based on a statistical approach.

### Multiple structural change analysis confirms that the RAM of the *mto2-2* mutant is devoid of the PD

**Fig. 3.**
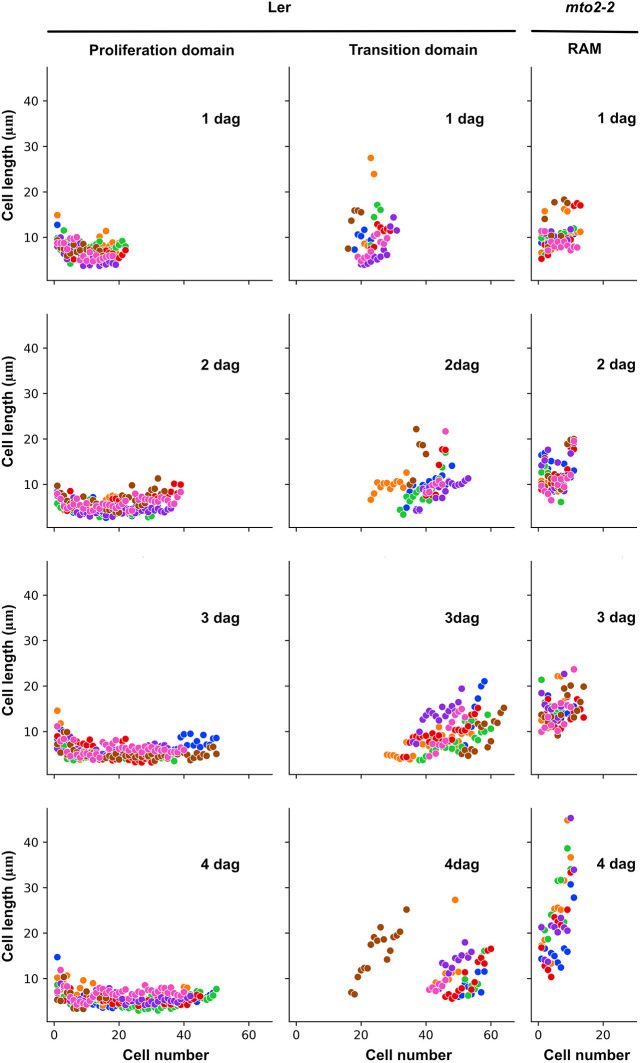
**Cell length profile in roots of Wt and *mto2-2* seedlings within the proliferation and transition domain of Wt and within the RAM of the *mto2-2* mutants.** For both genotypes, the boundaries between the domains or zones were identified by the MSC approach in one independent experiment (*n*=7). Note that the cell length distribution within the RAM in the mutants is similar to the transition domain of Wt plants. See also Fig. S5 for an independent experiment. Cell lengths for an individual root are marked with the same color.

The boundaries between the PD and TD and between the RAM and the EZ are not always obvious (Ivanov and Dubrovsky, 2013) and are frequently defined arbitrarily (Ivanov and Dubrovsky, 1997; Lavrekha et al., 2017). To define the boundaries between the domains or zones, we have previously proposed an approach for an objective non-arbitrary delimitation of growth boundaries in the growing root portion based on a multiple structural change algorithm (MSC) analysis (Pacheco-Escobedo et al., 2016), which detected that two is the most parsimonious number of breakpoints in Wt *A. thaliana* root apices, one between the PD and TD and another one between the RAM and the EZ. With this approach, we detected position of breakpoints (Fig. S4) and the whole RAM and its domains lengths expressed in µm, as well as the number of cells in each domain (Fig. 3; Fig. S5; Table S1). The number of transitions or breakpoints for the *mto2-2* mutant was detected to be zero, one, and two in 5, 66 and 29% of cases, respectively (*n*=76; Table S2). In most of the *mto2-2* roots (66%) one breakpoint, between the RAM and the EZ, suggested, based on cell length distribution, that the RAM was represented by only the TD (Table S1; Fig. 3; Fig. S5). In the *mto2-2* mutant seedlings of different ages, the algorithm detected the PD in only 21-32% of seedlings (*n*=19; Table S2). In these cases, cell length in the PD was 1.7-2.5 times longer than in the PD of wild type (*P*<0.001; Table S1), suggesting that these cells resembled the cells of the TD. This illustrates that the MSC algorithm is a rather stringent approach for determination of the domains or zones. Interestingly, the MSC algorithm also detected a small number of plants (5%) in which no single breakpoint was detected (Fig. S4), suggesting that all the cells in the apex steadily increased their length, and no RAM and EZ boundary was detected, apparently reflecting determinate root growth in the *mto2-2* mutant. Thus, in the *mto2-2* mutant, the PD was absent or negligible (Table S1). In the 1 DAG mutant, cell length in the TD was similar to that in Wt TD, and in 2-4 DAG seedlings, the cells were 10 to 40% longer than in Wt (Table S1). In conclusion, cell length profile analysis showed that the RAM in *mto2-2* mutant roots is composed of a single domain which behaves as the Wt TD and that the PD is absent in the mutant root.

### Endoreduplication in the *mto2-2* RAM supports that its cells belong to the TD

The RAM TD is characterized by rare cell proliferation events and the beginning of endoreduplication (Hayashi et al., 2013; Ishida et al., 2010; Pacheco-Escobedo et al., 2016; Vanstraelen et al., 2009). As both the mathematical modeling and MSC approaches suggest that the RAM of the *mto2-2* mutant behaves as the TD, we wanted to confirm this with a molecular marker. In *A. thaliana*, *CELL-CYCLE SWITCH 52 A1* (*CCS52A1*), an ortholog of yeast and *Drosophila Fizzy-Related 2* (*Fzr2*), is necessary and sufficient for the transition from mitotic to endoreduplication cycle and, in the root, is expressed starting from the TD of the RAM (Larson-Rabin et al., 2009; Pacheco-Escobedo et al., 2016; Vanstraelen et al., 2009).

**Fig. 4.**
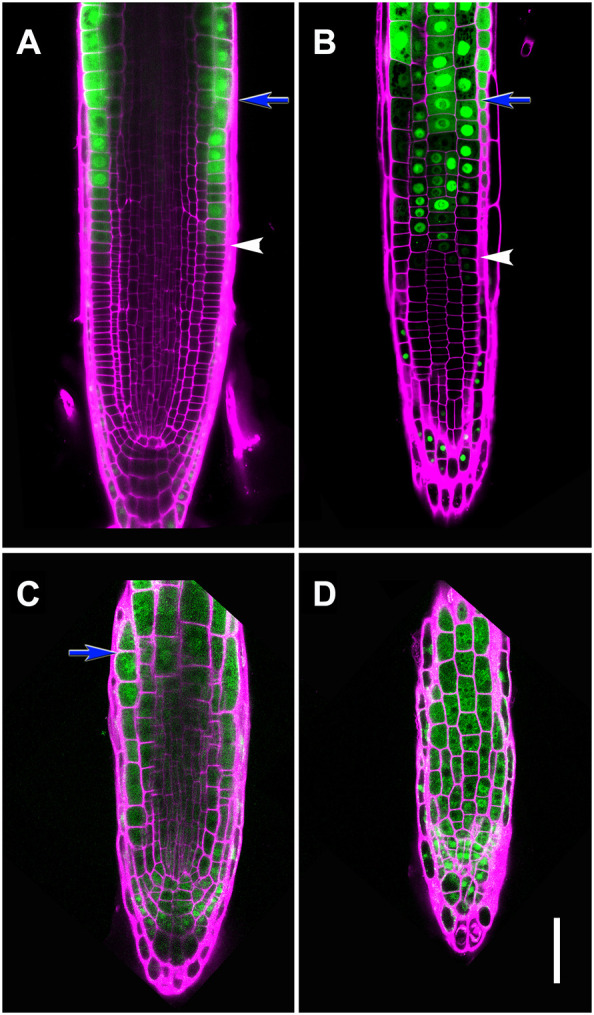
**Expression of the endoreduplication marker *pCCS52A1::CCS52A1-GFP* in Wt and the *mto2-2* mutant*.*** (A-D) Wt (A,B) and *mto2-2* mutant (C,D) seedlings at 3 DAG showing *CCS52A1* activity (green) and propidium iodide-stained (pseudocoloured in magenta) cell walls. (A,C) median confocal sections at the level of the QC. Note that in panel C, in the shootward direction the section becomes paradermal. (B,D) Paradermal (tangential) sections at the level of the epidermis. Arrows and arrowheads indicate a boundary between the RAM and the EZ and between the PD and TD, respectively; *n*=12 (Wt) and 18 (*mto2-2*). Scale bar: 50 μm.

We introduced endoreduplication marker *pCCS52A1::CCS52A1-GFP* (Vanstraelen et al., 2009) into the *mto2-2* mutant. As expected, in the Wt, *pCCS52A1::CCS52A1-GFP* is expressed exactly at the boundary between the PD and TD (Fig. 4). Consistent with reported *CCS52A1* expression pattern (Brady et al., 2007; Vanstraelen et al., 2009; Winter et al., 2007), we found the strongest expression in the epidermis, with a sharp decrease towards internal root tissues (Fig. 4). In 3 DAG *mto2-2*, when the RAM was still present, practically all the cells in the epidermis and cortex were expressing *CCS52A1* (Fig. 4), confirming that its cells were undergoing the endoreduplication cycle. Similar results were obtained on 2 DAG seedlings (*n*=9 for Wt and 6 for *mto2-2*; Fig. S6C).

To confirm this result, we transformed *mto2-2* with *pAtPCNA1::AtPCNA1-sGFP* (proliferating cell nuclear antigen-sGFP fusion) that shows speckled patterns of AtPCNA1 signal in late S-phase detected in both mitotic and endoreduplication cycles (Yokoyama et al., 2016). These authors showed that in the Wt mitotic cycle, whole S-phase duration is 3 h (Yokoyama et al., 2016). Time-lapse experiments (24 h, between 3 and 4 DAG) showed that this speckled pattern was maintained in the *mto2-2* RAM cells at least over a period ranging from 10 to 13 h (Fig. S6) and was accompanied with slow cell growth typical for the TD of the RAM. This suggests that the cells are in the endoreduplication cycle. The process of the RAM exhaustion is heterogeneous in time (Reyes-Hernández et al., 2019). When the *mto2-2* seedlings still possessed the RAM at 5 and 7 DAG, they were incubated for 24 h with 5-ethynyl-2′-deoxy-uridine (EdU) and it was found that the RAM cells had large nuclei that incorporated EdU even in the QC and stem cells. Both types of experiments suggest that the RAM cells switched to endoreduplication. Moreover, occasional divisions in the RAM, including stem cells, were also detected (Figs S6,S7).

Collectively this analysis showed that Thr synthesis is essential for establishment of the RAM PD, which is not established in the *mto2-2* mutant. The TD in the mutant root was similar to that in Wt. After some of its cells completed their cell division cycle they continued their slow growth and entered the endoreduplication cycle. Next, we investigated whether this unusual RAM organization with only a TD in the *mto2-2* mutant depends on auxin and known RAM maintenance pathway factors.

### The *MTO2* requirement for PD establishment is auxin-independent

Experiments described so far suggested that the PD and TD are uncoupled in the *mto2-2* mutant, implying that *MTO2* is required specifically for the PD establishment and SCN function. Auxin and a number of regulatory pathways are involved in the RAM PD maintenance (Blilou et al., 2005; Ioio et al., 2008; Moubayidin et al., 2013; Pardal and Heidstra, 2021). So, we aimed to describe how molecular markers related to these pathways operate in the *mto2-2* background in which only the RAM TD is present. We have previously shown that *mto2-2* root growth inhibition by exogenous auxin was similar to that in Wt, that auxin did not rescue the *mto2-2* phenotype and that the auxin response was progressively diminished in the mutant (Hernández-Barrera et al., 2011). Our later analysis showed, however, that by unknown reasons the cytoplasm in the root tip of the *mto2-2* mutant is much denser than in the Wt (Reyes-Hernández et al., 2019). Thus, if a fluorescent signal is not present in the mutant, it may be either absent or not detected due to light scattering. To resolve this dilemma, we used a Multiphoton Microscopy System (two-photon microscopy; 2P) that excites fluorophores at a greater depth within live root tissues. This approach showed that, when confocal laser scanning microscopy (CLSM) is used the signal present in a tissue can be not detected (Fig. S8).

**Fig. 5.**
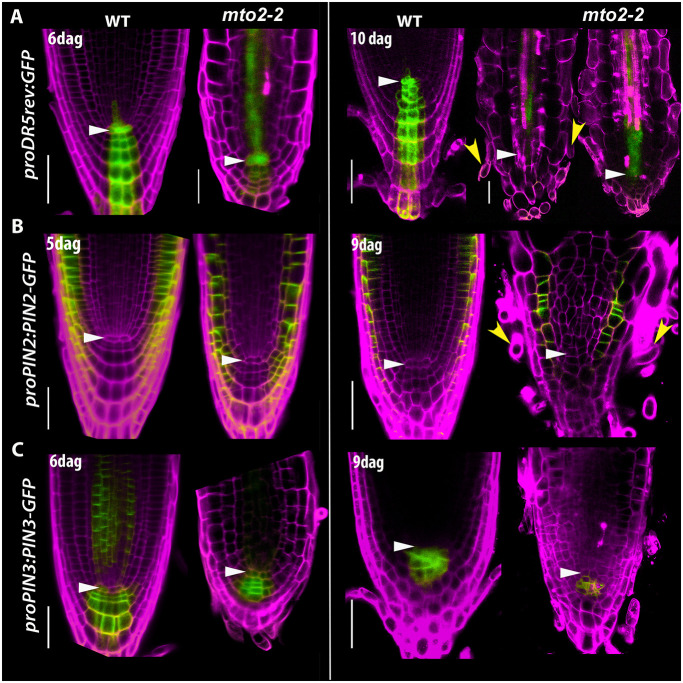
**Auxin response and expression of auxin efflux transporter markers during the *mto2-2* root apical meristem exhaustion.** (A) Expression of *proDR5rev:GFP* in *mto2-2* and Wt primary root. (B) Expression of *proPIN2:PIN2-GFP* in *mto2-2* and Wt primary root. (C) Expression of *proPIN3:PIN3-GFP* in *mto2-2* and Wt primary root. The left two columns (5-6 DAG seedlings) show images acquired with CLSM at stages when the root apical meristem in the *mto2-2* mutant is still present. The right columns show images acquired with 2P that led to visualization of developmental changes until the RAM exhaustion stage. In panel A, two 10 DAG apices of the mutant are shown, one with root hairs formed at the very tip and another without them. Propidium iodide-positive signal is pseudocoloured in magenta. White arrowheads indicate position of the QC; yellow arrowheads indicate root hairs. For each marker line, age and condition, *n*=5-7. Scale bars: 50 μm.

Analysis of *proDR5rev:GFP* auxin response marker expression (Friml et al., 2003; Ulmasov et al., 1997) showed that the maximum auxin response in the QC is maintained in the *mto2-2* mutant during RAM exhaustion and is preserved in the protoxylem cells (Fig. 5). We also examined expression of auxin-efflux transporter markers *proPIN2:PIN2-GFP and proPIN3:PIN3-GFP* in the *mto2-2* background. PIN2 and PIN3 are involved in redistribution of auxin in the RAM and, as such, are essential for maintenance of auxin gradients in the root (Křeček et al., 2009; Wiśniewska et al., 2006). The expression pattern and membrane localization of both *proPIN2:PIN2-GFP* and *proPIN3:PIN3-GFP* in *mto2-2* were apparently similar to that in Wt plants. Indeed, even at advanced stages of RAM exhaustion, PIN2*-*GFP was still detected in the epidermal and cortical cells (Fig. 5B).

When L-kynurenine, a potent inhibitor of the indole-3-pyruvic acid pathway of auxin biosynthesis, targeting the key enzymes encoded by *TRYPTOPHAN AMINOTRANSFERASE OF ARABIDOPSIS 1*, *TRYPTOPHAN AMINOTRANSFERASE RELATED 1* (*TAR1*) and *TAR2* (He et al., 2011) is applied, the *mto2-2* roots did not abolish the RAM exhaustion program (Fig. S9). Collectively these results suggested that, in roots lacking the PD, RAM exhaustion in *mto2-2* is not related to changes in auxin signaling, transport, or synthesis.

### The expression of RAM markers in the *mto2-2* RAM does not require the PD

**Fig. 6.**
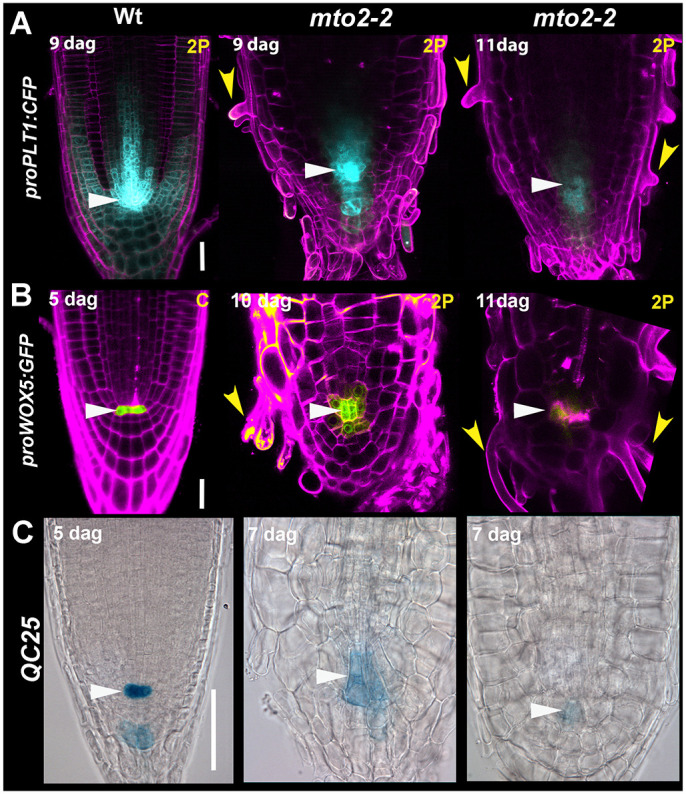
**Expression of PLT1 and the stem cell niche markers in the *mto2-2* background is maintained.** (A-C) *proPLT1:CFP* (A), *proWOX5:GFP* (B) and *QC25:GUS* (C) expression in the root apical meristem. The images were acquired using 2P (A,B), CLSM (Wt in B) or DIC (C) microscopy. White arrowheads indicate the QC; yellow arrowheads indicate root hairs. Propidium iodide-positive signal is pseudocoloured in magenta. For each marker line on A and B and for each age, *n*=5-10: for C *n*=11 (Wt) and 39 (*mto2-2*) in at least two independent experiments. Note that in the *mto2-2* mutant, the roots show complete exhaustion of the root apical meristem. Scale bars: 25 μm.

Previously we have shown that expression of enhancer trap *J1021* (marker of the root cap), *proWOX5:GFP* and *proSCR:GFP* are maintained in the *mto2-2* root, but become dimmer during RAM exhaustion (Hernández-Barrera et al., 2011). Here, we analyzed several RAM maintenance markers using 2P. Our analysis showed that, at advanced stages of RAM exhaustion, in most cases the *proPLT1:CFP, proWOX5:GFP* (Fig. 6A,B; Fig. S8) and *proSCR:GFP* (Fig. S10) expression was still detected.

The expression pattern of pro*PLT1:CFP* in *mto2-2* shows that it is maintained at high levels in the QC cells even at advanced stages of RAM consumption (Fig. 6A). A QC marker, QC25 (Sabatini et al., 2003), was expressed in the *mto2-2* mutant similar to Wt. Under our growth conditions, in 10 out of 11 Wt 5 DAG seedlings, *QC25:GUS* expression was detected (Fig. 6C). In the *mto2-2* seedlings of the same age, all of them (*n*=10) showed GUS expression, but at 7 DAG, when RAM exhaustion was in progress, only 31% of seedlings maintained GUS expression (*n*=29), further suggesting that during RAM exhaustion the QC identity was compromised. Indeed, we found that during RAM exhaustion the *mto2-2* QC cells divided (Fig. 6B, 10 DAG) once (8 out of 20 roots) or twice (6 out of 20 roots) in 5-10 DAG seedlings.

Overall, this analysis suggests that while the RAM is not completely exhausted, the expression of known genes involved in its maintenance is not compromised in the *mto2-2* root. Thus, expression of the studied markers does not depend on presence of the RAM PD. In addition, this suggests that expression of these markers is not sufficient to establish the RAM PD. These data collectively indicate that the Thr-dependent mechanism of RAM maintenance may act independently of the known regulatory pathways of RAM maintenance. Importantly, despite the fact that the *mto2-2* RAM cells enter endoreduplication, some of them are still capable of cell division (Figs S6,S7), which was also shown with *CycB1;1DB::GFP* expression (Hernández-Barrera et al., 2011). Altogether these results suggest that the RAM represented in the mutant with the TD is functional, with some cells progressing through a mitotic cycle and some entering endoreduplication.

As both available hypomorphic mutants in a gene encoding TS1, *mto2-1* and *mto2-2*, show a consistent phenotype of RAM exhaustion (Reyes-Hernández et al., 2019), and the present study showed that the RAM PD is not established, we asked where, theoretically, Thr is more essential in Wt seedlings: in the SCN, in the whole PD of the RAM, or in both? To address this question, we implemented *in silico* modeling with an attempt to predict free Thr distribution based on the gene expression pattern of the known genes directly involved in its metabolism. Current methods do not permit us to answer this question in an experiment at a cellular or tissue level.

### *In silico* modeling of Thr distribution predicts that Thr is essential for stem cell function

The results presented so far point to a central role of free Thr for RAM maintenance that is severely disturbed in the *mto2-2* mutant, leading to the RAM exhaustion. Nevertheless, it remains undetermined where exactly in the root apex is Thr essential, specifically in the whole RAM or in the SCN. To address this, we explored two alternative models of Thr distribution in the RAM: a simplified model of Thr distribution along the root based only on its synthesis and an extended model that also considers the impact of Thr catabolism in the RAM.

**Fig. 7.**
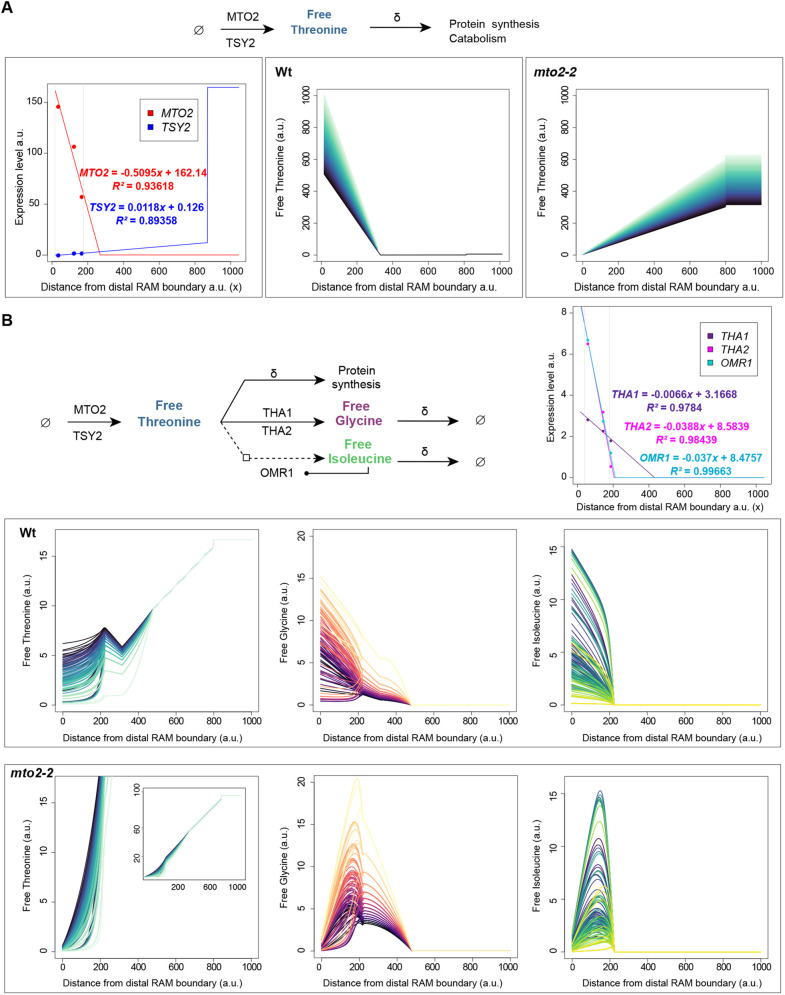
**Plausible models of Thr distribution in *A. thaliana* root.** (A) Model of Thr synthesis in the root considering the role of MTO2 and TSY2. The plots show the expression pattern of *MTO2* and *TSY2* along the root (*x*-axis) derived from linear functions based on expression data from Wendrich et al. (2017) (grey lines indicate the region in the *x*-axis that corresponds to the RAM) and the predicted free Thr distribution in Wt and *mto2-2* roots. A total of 10,000 parameter sets were used to solve the ODE; each color is a different parameter set. (B) Model that combines Thr synthesis and catabolism in the root considering the role of MTO2, TSY2, THA1, THA2 and OMR1. The plots show the expression of the genes involved in Thr catabolism and, below, the free Thr, free Gly and free Ile distribution for Wt and *mto2-2* roots. Importantly, Gly and Ile represents the pool of free amino acids produced via Thr catabolism. For *mto2-2*, the inset shows the extent of the increase in the levels of free Thr (notice the different scales of the *y*-axis). A total of 125,000 parameter sets were used to solve the ODE (Fig. S11), and only a fraction (4000) is shown here for clarity.

For the first model based on Thr synthesis we used the previously introduced ODE that describes Thr synthesis mediated by MTO2 and TSY2, and the constant usage of Thr for protein synthesis and catabolism (see Materials and Methods). This ODE was used to predict the Thr level in each cell of a one-dimensional row of cells (*x*-axis in plots in Fig. 7A) with varying levels of expression, and thus of activity of the enzymes MTO2 and TSY2. It is currently impossible to measure free Thr levels at a cellular resolution in the root, and therefore these data do not exist to fit the model parameters. As an alternative, we ran simulations over a wide range of parameter values to describe the possible behavior of the system (i.e. predicted patterns of Thr distribution along the root). The Wt simulations with the different parameter sets all yielded free Thr distributions with the highest levels at the SCN (distal RAM boundary), which gradually decrease along the RAM and plummet in the EZ to low, but higher than zero, levels (Fig. 7A). Different parameter sets resulted in variations in the magnitude of the free Thr distribution, but its overall shape is conserved in all of them. In contrast, simulations for the *mto2-2* mutant yielded distributions where the free Thr levels are drastically diminished in the SCN, whereas the rest of the RAM is also affected but to a lesser extent (Fig. 7A). Importantly, in the *mto2-2* simulations, the cells representing the EZ and early onset of differentiation show an increase in free Thr levels as a result of the compensation by TSY2 activity in the mutant. This causes overall higher free Thr levels in *mto2-2* roots compared with Wt roots, but a deficiency of free Thr in the SCN region as *TSY2* is not highly expressed in these cells (see also Fig. S3). This is consistent with measurements of free Thr in Wt and *mto2-2* that have shown that Thr content in roots of *mto2-2* is actually 59% higher than in Wt (Reyes-Hernández et al., 2019). The model suggests that, despite this higher free Thr content, root growth is severely compromised in *mto2-2* because of Thr deficiency in the SCN. The perturbed distribution pattern predicted for *mto2-2* is observed over a wide range of parameter values revealing it as a general pattern with a limitation of Thr synthesis in the SCN. These simulations considering only the role of Thr synthesis strongly suggest that the SCN is a region of high Thr synthesis in the root, and the most affected upon the reduction of the activity of MTO2 in the *mto2-2* mutant.

Next, we explored the role of Thr catabolism in the root using the mathematical model (Fig. 7B). It has been proposed that Thr catabolism might be a crucial regulator of stemness in plants similar to animals (Sahoo et al., 2021). Namely, Thr catabolism plays an essential role in the maintenance of stemness in animal embryonic stem cells (Wang et al., 2009), where it has been suggested to be a source of purines for DNA synthesis and of S-adenosylmethionine for epigenetic modifications via DNA and histone methylation (Sahoo et al., 2021; Wang et al., 2009). Although the role of Thr catabolism in plant SCNs remains to be established, it is remarkable that several of the enzymes involved in Thr catabolism are highly expressed in the root SCN and their expression gradually decreases as cells approach the EZ (Wendrich et al., 2017), a pattern reminiscent of that described for catabolic factors in embryonic animal stem cells (Wang et al., 2009). Thus, we extended the mathematical model to address the potential role of Thr catabolism in the Thr-dependent mechanism underlying RAM maintenance. The enzymes mediating Thr catabolism in plants are THREONINE ALDOLASE 1 (THA1) and THA2, which catalyze the conversion of Thr to glycine (Gly) and acetaldehyde, and L-O-METHYLTHREONINE RESISTANT 1 (OMR1), a deaminase/dehydratase that participates in the production of isoleucine (Jander and Joshi, 2009; Joshi et al., 2006). These enzymes were included in the model through a modified ODE for free Thr estimations with explicit terms for the catabolism of Thr mediated by THA1/2 enzymes and by OMR1 (see Materials and Methods). We also included two additional ODEs to describe the free Gly and free Ile derived from Thr catabolism and considered the negative feedback regulation of OMR1 by Ile (Xing and Last, 2017). The spatial distribution of Thr synthesis and catabolism in the root was explored by solving the ODEs in each cell (*x*-axis in plots Fig. 7B) with varying levels of expression of these enzymes along the root based on previously published data (Wendrich et al., 2017). As with the previous ODE model, we ran the simulations using a wide range of parameter values to explore the possible behaviors of the system.

The extended model for Wt predicted relatively low levels of free Thr in the SCN, yet still higher than zero, followed by a gradual increase throughout the RAM and EZ, and then a sudden decrease. This predicted distribution is in stark contrast to the one from the previous model, specifically for the SCN. However, there is no contradiction as both models predict the SCN as an active site of Thr synthesis, although the second model complements this prediction by revealing it is also a site of active catabolism, resulting in very low levels of free Thr (Fig. 7B). Consistently, Gly and Ile derived from Thr catabolism are predicted to have the highest levels in the SCN region. In the case of the *mto2-2* simulation, the model predicts that the levels of free Thr, and Gly and Ile (derived from Thr catabolism) are all severely affected in the SCN, but at a lesser extent in the rest of the RAM. The free Thr reaches very high levels in the EZ and early DZ, while levels of Gly and Ile, derived from Thr catabolism (based on expression patterns of *THA1*, *THA2* and *OMR1*), decrease similar to those predicted in the Wt. This is consistent with experimental data that has established that whole root free Thr levels are significantly increased in the *mto2-2* roots, but not Gly and Ile levels (Reyes-Hernández et al., 2019). The simulation predicts that in the case of Gly and Ile (derived from Thr catabolism) the overall levels of these free amino acids might not be drastically affected in the root, but it is their distribution that might be notably changed, maintaining the lowest levels in the SCN cells. To further assess the role of Thr catabolism in RAM maintenance, we implemented this extended ODE model to simulations of the growing root. Importantly, considering both Thr synthesis and catabolism, the mathematical model predicted cell length distributions in Wt and the *mto2-2* mutant similar to those shown in Fig. 2, and thus recovered indeterminate and determinate root growth in Wt and the *mto2-2* mutant, respectively (Fig. S12). Overall, these simulations suggest that the coordinated activity of the enzymes directly involved in Thr synthesis (MTO2 and TSY2) and catabolism (THA1, THA2, OMR1), results in the root SCN being the region of highest Thr synthesis and catabolism in Wt roots, and the region most affected in *mto2-2* roots. Notably, the rest of the RAM is still affected but not as much as the SCN. Thus, addition of Thr catabolism recovers the same output results as the model considering only Thr synthesis (Fig. 2), showing the importance of both processes in determination of growth pattern.

Overall, the model simulations reveal that the expression pattern of the enzymes involved in Thr synthesis and catabolism yield a compartmentalization of metabolic activity along the root that is severely disturbed in *mto2-2*. Based on these results we propose that both Thr synthesis and catabolism are crucial for RAM organization and the SCN state, suggesting that a deficiency of Thr in the SCN is the underlying cause of the *mto2-2* phenotype. This is similar with what has been observed in animal stem cells and poses Thr and its metabolism as a fundamental regulator of stemness in plants (Sahoo et al., 2021; Wang et al., 2009).

## DISCUSSION

In both plants and animals, fundamental principles of tissue generation depend on the activity of stem cells that produce transit-amplifying cells, which subsequently transit to cell differentiation (Dubrovsky and Ivanov, 2021; Zhang and Hsu, 2017). This tissue organization is essential for regeneration and growth. The transit-amplifying cells, occupying a transitional stage between stem cells and the final differentiated stage, are essential for both stem cell maintenance and transition to differentiation in plants (Barlow, 1978; Efroni et al., 2016) and animals (Beumer and Clevers, 2021; Kurosaka et al., 2011; Zhang and Hsu, 2017). At the same time, transit-amplifying cells lose their proliferation potential gradually. For example, inactivation of a β-catenin gene in dermal papilla of a hair follicle causes a decrease in proliferation of transit-amplifying cells, promoting the transition to the final differentiation stage (Enshell-Seijffers et al., 2010). Similarly, in plants, cells in the proximal RAM portion (TD cells) gradually diminish their proliferation activity, as can be visualized with *CyclinB1;1_DB_:GFP-*marked cells occasionally found in the TD of the RAM (Pacheco-Escobedo et al., 2016). As the cells in this region are still capable of division, they belong to the RAM portion and constitute its TD. Thus, transit-amplifying cells constitute both domains of the RAM, but in the TD not all cells have sufficient time to complete their cell division cycle and start endoreduplication (Hayashi et al., 2013; Pacheco-Escobedo et al., 2016). As rapid elongation in the EZ takes place simultaneously in all cell types, a challenge is to establish a boundary between the PD and TD, not between the RAM and EZ. Arbitrarily, this boundary is defined based on cell length changes; a location where cells start to become longer than the dividing cells but do not yet elongate rapidly determines the PD-TD boundary. To decrease a subjectivity factor in the determination of this boundary, the MSC algorithm was adapted to cell length profile analysis (Pacheco-Escobedo et al., 2016) and, in this work, it is further improved (see Materials and Methods).

The results presented here have shown that the PD and TD can be uncoupled by a mutation in the *MTO2* gene and the whole RAM in the mutant is represented by only a TD, indicating that the RAM cells can be in two alternative cell states (PD or TD) and therefore the TD is not simply a boundary between the RAM and the EZ. This conclusion is supported by three lines of evidence: (1) Cell length profile and the MSC algorithm showed absence of the PD in the RAM of the majority of the *mto2-2* roots; (2) *in silico* root modeling predicted that the mutant RAM is devoid of cells with PD characteristics; (3) the whole RAM in the mutant behaves as a TD of the Wt, showing high proportion of the cells undergoing endoreduplication. Thus, as the TD and PD can be uncoupled, the TD, called also ‘transition zone’, is not part of a separate or independent root zone as sometimes considered (Baluška and Mancuso, 2013; Baluška et al., 2010; Takatsuka et al., 2018), but imminently a part of the RAM. The term ‘transition zone’ is also used for a boundary between the proximal portion of the TD of the RAM and the EZ, the setup of which is important for the beginning of rapid cell elongation and depends on coordinated auxin and cytokinin signaling (Di Mambro et al., 2017; Pacifici et al., 2018; Salvi et al., 2020a,b). Interestingly, at this boundary an abrupt fall in free Thr level is predicted following the gradual decrease in the expression of *MTO2* (Fig. 7B).

Cells in the TD of the RAM grow at a relatively low rate, similar to cells in the PD, but divide less frequently (Ivanov and Dubrovsky, 2013), thereby attaining a greater length (Table S2). Importantly, the relative rate of cell growth in the TD is the same as in the PD (Hernández-Herrera et al., 2022; van der Weele et al., 2003), consistent with the longer cells found in the RAM of *mto2-2*. Even though the RAM in the *mto2-2* mutant is represented by only the TD cells, expression of classical transcription factors involved in RAM function (Fig. 6) and auxin signaling and transport (Fig. 5) in the *mto2-2* root are maintained, underlying a relative autonomy of the Thr-dependent pathway of the RAM maintenance. Here, we show that establishment of the PD is compromised when Thr metabolism is affected. Our previous study suggested a cellular mechanism dependent on QC activation during the process of RAM exhaustion in the mutant affected in Thr synthesis (Reyes-Hernández et al., 2019). Thus, on one side Thr is required to maintain cell proliferation and establish the PD of the RAM, and on the other side it maintains quiescence of the QC and stem cell activity. As *pCCS52A1::CCS52A1-GFP* in the *mto2-2* mutant is expressed in the ground tissue and columella stem cells (Fig. 4), the question arises whether these stem cells pass through at least one cell division cycle before entering endoreduplication. Among all *mto2-2* roots imaged with the various approaches described (*n*>210), only one root was found where stem cells divided (Fig. S7), suggesting that proliferation of the QC cells (Fig. 6B) is accompanied by an arrest in stem cell activity.

Through our computational framework, we further identified the SCN as the most perturbed region in *mto2-2* roots, pinpointing the zone in the RAM where free Thr might be indispensable to drive indeterminate growth. This is a nice example of how, through integrative experimental and computational approaches, we can accelerate the discovery of the mechanisms underlying new regulatory pathways in the RAM, providing explanations to current data and inspiring new experimental endeavors. The crucial role of Thr in stem cell maintenance across the animal and plant kingdoms is intriguing (reviewed by Sahoo et al., 2021). Mouse embryonic stem cells transit to differentiation when deprived of Thr (Wang et al., 2009), similar to the SCN cells in the *mto2-2* mutant. Importantly, the *in silico* root model was able to reproduce this developmental pattern based entirely on the predicted Thr distribution along the root (Fig. 2).

Through years of research, important regulatory pathways involved in the RAM maintenance, with the PLT transcription factors being at the center stage, were discovered (Mähönen et al., 2014). Notably, the expression of *PLT1* is still detected in the SCN in *mto2-2* roots, indicating that the novel Thr pathway we report here is apparently independent of the PLT pathway. A remaining challenge will be to uncover how these pathways are interrelated, as the metabolic state has been shown to influence the stability of PLT2 (Zhang et al., 2022). Experimental and computational approaches implemented here will be fundamental to uncover how the metabolic state regulates SC activity. It can also help to understand the RAM domains in other mutants with determinate root growth to establish similarities and differences in the mechanisms driving their dramatic root growth alterations.

Part of stem cell progeny represents transit-amplifying cells, and these divide several times and eventually become differentiated cells. This continuum represents a developmental sequence from the least differentiated cell state to the final cell state. These states are longitudinally patterned in the plant root. Thus, prediction of free Thr longitudinal distribution can help to understand the Thr-dependent mechanism of the RAM maintenance. Here, to the best of our knowledge, we created the first *in silico* model of an amino acid distribution along a developing organ in plants. Importantly, this model was able to reproduce experimental results of relative Thr distribution in the mutant roots (Fig. 7). Based on Thr synthesis only, the model predicts the highest Thr level in the SCN of Wt roots (Fig. 7A). Incorporation of Thr catabolism suggests a fine tuning of free Thr level in this compartment and gradual increase of free Thr along the Wt RAM. Considering both Thr synthesis and catabolism in the mutant RAM, the lowest Thr level is predicted to be in the SCN (Fig. 7). This can explain why, in the *mto2-2* mutant, the QC cells lose their quiescence and the RAM becomes exhausted. The *in silico* modeling also showed that Thr catabolism appears to be an important factor in stem cell maintenance. This aspect of the possible mechanism of the root SCN maintenance is similar to that found in animal systems (Alexander et al., 2011; Wang et al., 2009). Overall, a compartmentalization of metabolic activity along the root revealed by *in silico* modeling emerged as an important factor affecting developmental scenarios. How this compartmentalization is maintained needs to be established.

## MATERIALS AND METHODS

### Plant material and growth conditions

The *mto2-2* mutant is in the Ler background. The enzymes directly involved in Thr synthesis in *A. thaliana* are TS1 and TS2, which are encoded by *MTO2* (*AT4G29840*) and *AT1G72810*. By convention a gene name should contain three letters [Meinke and Koornneef (1997) and https://www.arabidopsis.org/portals/nomenclature/guidelines.jsp], so we called the latter gene *TSY2* and the respective mutants *tsy2* (*ATS* abbreviation is already taken)*.* If the community feels appropriate, *MTO2* gene and TS1 protein (Bartlem et al., 2000; Curien et al., 2009) can be renamed to *TSY1* and TSY1, respectively, but here we maintain the name of MTO2 for threonine synthase 1 (Bartlem et al., 2000).

The *mto2-2* mutant, *pMTO2:MTO2-GFP* in the *mto2-2* background (Hernández-Barrera et al., 2011; Reyes-Hernández et al., 2019), and transgenic lines *proWOX5-GFP* (Sarkar et al., 2007), *proSCR:GFP* (Heidstra et al., 2004), *proPLT1:CFP* (Galinha et al., 2007), *proDR5rev:GFP* (Friml et al., 2003), *proPIN2:PIN2-GFP proPIN3:PIN3-GFP* (Friml et al., 2002), QC25, (Sabatini et al., 2003), *J0121* (Laplaze et al., 2005) and *pCCS52A1::CCS52A1-GFP* (Vanstraelen et al., 2009) have been previously described. The pGWB504 binary vector carrying *pAtPCNA1::AtPCNA1-sGFP* (Yokoyama et al., 2016) was kindly donated by S. Matsunaga (Tokyo University, Japan) and was used for floral deep transformation of Ler Wt and *mto2-2* plants.

Seedlings were grown as previously described (Reyes-Hernández et al., 2019). Briefly, they were grown in 0.8% agar 0.2× MS medium supplemented with 1% sucrose under a 16 h photoperiod with a light intensity of 105 μmol photons m^–2^ s^–1^. For pharmacological treatments, 2 DAG seedlings grown in 0.2× MS medium were transferred to media supplemented with kynurenine and grown for additional 9 days at the concentrations indicated. Stock solutions of 5 mM kynurenine were prepared in DMSO.

### Histochemistry and microscopy

For GUS staining, the plants were incubated in 90% (v/v) ice-cold acetone at −20°C for 30 min, stained for 16 h as described by Hemerly et al. (1993) and cleared. For clearing, roots were fixed and mounted in an NaI-based clearing and mounting solution (4.2 M NaI and 8 mM Na_2_S_2_O_3_ in 65% glycerol and 2% DMSO) as previously described (Dubrovsky et al., 2009) with modifications (Hernández-Barrera et al., 2011). Because the *mto2-2* mutant had a very dense cytoplasm, for cell length profile data collection a different clearing protocol was implemented. First, roots were fixed in freshly prepared fixative as described in Ilina et al. (2012) with modifications [4% paraformaldehyde, 0.1% Triton X-100 in 0.3× microtubule-stabilizing buffer (MTSB)] overnight at 4°C. MTSB contained 50 mM 1,4-piperazinediethanesulfonic acid (PIPES), 5 mM MgSO_4_ and 5 mM EGTA. After fixation, roots were rinsed with 0.3× MTSB for 5 min. Then MTSB buffer was replaced with 30% glycerol (v/v) prepared in 2% DMSO (v/v) and roots were incubated for at least 1 h (commonly 24 to 72 h) at room temperature. Subsequently, roots were mounted in NaI-based clearing and mounting solution described above. Roots were left in this solution before observation for another 24 h. This protocol was named the MTSB-NaI-based clearing protocol. Details of the mounting procedure can be found in Napsucialy-Mendivil and Dubrovsky (2018). Photographs of cleared roots were taken with a Photometrics CoolSNAPcf Color Camera (Valley International Corporation).

To collect cell length profile data, two independent experiments were performed. To establish exact time after seed germination in both experiments seeds were plated as described and, starting from 30-36 h after plating, each seed was monitored for germination, which was recorded as the time the radicle protruded the seed testa (time 0 DAG). For the first experiment, at the times indicated the seedlings were fixed and cleared using the MTSB-NaI-based clearing protocol. The cells were measured directly from cleared preparations with an ocular micrometer using an Olympus BX 51 bright field microscope equipped with differential interferential contrast (DIC; Nomarski) optics. For the second experiment, the seedlings were fixed in 50% methanol and 10% acetic acid at 4°C for 5 h and stained with pseudo-Schiff as previously described (Truernit et al., 2008). Then, the samples were mounted in NaI-based clearing and mounting solution (Dubrovsky et al., 2009) and imaged using a Thorlabs confocal system, described below, built around a Zeiss Axiovert 200M microscope equipped with a C-APO 63×1.2NA W objective. Several images were collected for a single root, and then images were stitched using Pairwise Stitching plugin (Preibisch et al., 2009) of the open-source image processing package Fiji (ImageJ; Rueden et al., 2017; Schindelin et al., 2015). To measure cell lengths in the second experiment, a Cell_Length_V2.02 tool (http://www.ibt.unam.mx/labimage/proyectos/arabidopsis) was used as previously described (Pacheco-Escobedo et al., 2016). In both experiments, cell length profiles were obtained measuring cortical cell length starting from a cortex or a cortex-endodermis initial cell, and then the length of each subsequent cell in a cell file was measured.

Live or fixed roots were analyzed with a confocal system consisting of: a high-speed galvo-resonant scanner designed for visible (SCAN-VIS) wavelengths; a motorized pinhole wheel with round pinholes; 488 and 405 nm laser sources; 495 nm and 562 nm longpass dichroic 440/40, 525/50 and 630/92 nm bandpass emission filters for DAPI, GFP and propidium iodide (PI) fluorescence, respectively; PMT modules (standard sensitivity); a linear motor travel *xy* stage and a *z*-axis piezo stage with controllers; and ThorImageLS 4.0 software (all from Thorlabs). For 2P and mixed 2P and confocal mode, images were acquired using an Olympus FV1000 confocal on an Olympus BX61WI upright microscope equipped with XLPlan N 25× N.A. 1.05 W MP and a MAI TAI 2P laser. For confocal acquisition, GFP protein was excited with a 490 nm diode laser (Vortran Stradus^®^ 488, 75 mW), PI was excited with a 635 nm laser (Olympus built-in system). Emission was selected by BA530-545 and BA570-670 filters for GFP and PI, respectively. The 2P imaging was performed including the oil-immersion condenser for the XLPlan N 25× N.A. 1.05 W MP. GFP and PI were excited using a MaiTai Deep See eHP Ti:Sa 2P laser (Spectra Physics) locked to 900 nm. Emission was collected in non-descanned 2P mode by the FV10-MRG/R filter set using BA495-540 nm for GFP and BA575-630 nm for IP. Acquisition parameters and hardware configuration were defined using FV10-ASW and MAI TAI software. Laser power and PMT parameters were identical for all samples.

For analysis of cells passing through S-phase of the cell cycle, 5 and 7 DAG seedlings were transferred to plates with fresh growth medium supplemented with 10 μM 5-ethynyl-2′-deoxy-uridine, EdU (the EdU-Click-iT kit, Invitrogen-Alexa Fluor 594) and incubated for 24 h. Seedlings were fixed with 3.7% formaldehyde in phosphate-buffered saline (PBS) during 15 min at room temperature and material was processed in accordance with the manufacturer instructions except that instead of Hoechst 33342 we used DAPI at 5 µg ml^−1^. To identify mitotic figures, some roots were also fixed in 4% formaldehyde in 1× PBS solution for 60 min under vacuum. Then, the samples were washed with 1× PBS solution twice, 5 min each, under gentle shaking and then transferred to 1 μg ml^−1^ DAPI in 1× PBS for 1 h, in darkness, under vacuum. Finally, the samples were washed twice, 5 min each, under gentle shaking, and mounted in PBS for further analysis with CLSM. Relative GFP intensity was analyzed using Image J (Schindelin et al., 2015) after subtraction of the background GFP signal.

### Multiple structural change analysis

Cell length profile data were subjected to MSC analysis described in detail in Pacheco-Escobedo et al. (2016) and available at http://www.ibiologia.com.mx/MSC_analysis_ver2/. For this, the estimation of the most parsimonious model of the number of breakpoints for each cell length profile was detected using the Bayesian Information Criterion (BIC) (Fig. S4) using the breakpoints function of the R ‘strucchange’ package for testing structural changes in linear regression models (Zeileis et al., 2003, 2002). The package is available at The Comprehensive R Archive Network at https://cran.r-project.org/. Cell length profile data were collected from median sections similar to those shown in Fig. 1A. The primary roots in the *mto2-2* mutant and their apical meristems were shorter than in the Wt and frequently the roots were curved. Therefore, the number of cells in a profile was variable for different roots and was lower in the mutant than that in the Wt. Because the number of cells in a cell length profile in Ler and *mto2-2* roots differed considerably and cell lengths in roots of each genotype were also different (Fig. 1B), to perform the MSC analysis, we introduced the fraction value *f* for each genotype and in each experiment, which was defined as the minimal fraction of the number of cells describing a cell length profile in a sample of roots that can be part of a longitudinal domain or zone. The *h* value for each individual root was introduced as the minimal number of cells in a root that can constitute a longitudinal domain or zone; it is calculated as *h*=f*n, where *n* is the total number of cells in a cell length profile of an individual root. As MSC models are composed of lineal regression segments, the *h* value must be greater than the number of regressors of a lineal model (*y_0_* and the slope of the fit line) and smaller than half of the total number of cells in a profile, then 3≤*h*_*i*_<*n*/2. To calculate *h*, we determined the value of *f* (as the one that satisfies the condition that f*n_*s*_≥3, where *n*_*s*_ is the number of cells in the smallest cell length profile of a sample) for each genotype and experiment (Table S3). Based on the smallest cell length profile in a sample of roots, *f* values corresponding to the first experiment were equal to 0.12 (Ler) and 0.24 (*mto2-2*) and those corresponding to the second experiment were equal to 0.13 (Ler) and 0.24 (*mto2-2*) (Table S3). When *h* value was determined, the MSC analysis was performed. Commonly, in a steady-state growing Wt root, one breakpoint is found between the RAM and the EZ, and another breakpoint is detected between the PD and TD (Pacheco-Escobedo et al., 2016). Therefore, in this study for all Wt roots, a two breakpoints model was applied. When only one breakpoint was detected in the *mto2-2* mutant (Table S2; Fig. S4), this breakpoint referred to the boundary between the RAM and the EZ. In all these cases the whole RAM was treated as the TD because, on average, cell length in the RAM was similar or greater than that in the TD of the Wt seedlings (Table S1). To analyze the characteristics of the PD and TD of the RAM in Wt and the *mto2-2* mutant identified by the MSC algorithm, each parameter obtained for the mutant was compared with that of Wt root at the same age (Table S1).

### RNA extraction and quantitative RT-PCR

Total RNA was extracted from roots of Wt and *mto2-2* 4 DAG seedlings. The root portions of 0-1 and 1-4 mm from the at least 300 root tips were excised with a razor blade. RNA extractions were performed using the NucleoSpin RNA kit (Macherey-Nagel) according to the manufacturer's instructions. First strand cDNA was synthesized from 1 µg of total RNA using Superscript II Reverse Transcriptase (Invitrogen) according to the manufacturer's instructions; 50 ng of cDNA was used for each reaction. Quantitative RT-qPCR reactions were performed in triplicate for each of the two biological replicates of both Wt and the mutant using a LightCycler^®^ Nano Instrument (Roche). Two reference genes, *UBQ10* (*At4g05320*) and *EF1ALPHA* (*AT5G60390*), were used (Czechowski et al., 2005), and normalized expression levels were calculated according to Vandesompele et al. (2002). The primers used are shown in Table S4.

### *In silico* root model

A model of an *in silico* growing root was developed with a lattice-based approach, such that each cell is defined as a collection of grid points, the position of which are updated to consider the displacement that takes place due to growth of the cells at the root tip. Different root cell types are modeled: the epidermal, cortical, endodermal and stele cells, which are color-coded. To study the general effect of the Thr-dependence of cell proliferation during root growth, the developmental zones of the root were defined based on the position of the cells in such a manner that the length of each zone is proportional to those in *Arabidopsis* seedlings, similar to other studies (Shimotohno et al., 2015; van den Berg et al., 2021). The meristem organization is considered as reviewed by Dubrovsky and Ivanov (2021) and consists of a PD and a TD; in both domains cells grow slowly and longitudinally by adding a layer of new grid points at each simulation step, and cell division only takes place in the PD when cell size is doubled during a cycle time (from one division when a cell is born to the next one). The proximal stem (initial) cells also grow and divide, albeit at a lower rate than other cells in the RAM PD. The divisions of the proximal initials are asymmetric, producing a shootward daughter cell with a finite capacity for cell division (maximum of five cycles) and a rootward daughter cell that remains as a stem cell and thus has unlimited division capacity. The model assumes that cell division of the proximal initial cells and other RAM cells is allowed as long as the free Thr cell levels are maintained above a threshold. The cells grow rapidly in the EZ and stop elongating once they reach a maximal cell length. The parameters used to model cell growth dynamics were chosen to represent real roots. The model used a threshold cell length of dividing cells, extent of cell elongation in the EZ and length of fully differentiated cells. The conditions considered for the default simulation were ∼40 cells in the longitudinal axis of the RAM (32 in PD and 8 in TD) and 7-10 cells in the EZ. In the model, the growth dynamics of the distal (for the columella) and lateral (for epidermis and lateral root cap) initial (stem) cells, and of the cells of the columella itself, are not included. The cell lengths at each time step of the simulation result from the growth and proliferation processes modeled, particularly driven by free Thr as a hypothetical regulator of cell proliferation in the RAM. Simulations of both Wt and the *mto2-2* mutant have the same initial condition, as the *mto2-2* embryo radicle is unaffected compared with that of Wt (Hernández-Barrera et al., 2011) and the RAM cells of 0 DAG *mto2-2* seedlings are of the same length as in Wt (Fig. S1A).

To compare the cell lengths distribution of the RAM cells in *mto2-2* and Wt (PD and TD) roots at a specific time, we extracted the grid coordinates in the longitudinal axis of each cell to extract their cell lengths. Then, we compared the *mto2-2* RAM cell length distribution with that of Wt PD and Wt TD cells for as long as *mto2-2* roots grew (from *t*=1 to *t*=5 a.u.). For this we used a non-parametric Mann–Whitney *U*-test (given no normal distributions) and an unpaired two-tailed *t*-test (due to the large number of cells in each distribution). In both cases we found a temporal window where the cells in the TD in Wt are statistically indistinguishable from those of the *mto2-2* RAM. In Fig. 2C (inset) we show the result of the comparison for *t*=3.4, where *t_1_* and *t_2_* indicate the time range at which Wt TD cells and *mto2-2* RAM cells are statistically indistinguishable according to the Mann–Whitney *U*-test.

The results reported here are robust despite variations in the parameters controlling cell growth and gene expression. Regarding the former, we performed simulations with different cell length thresholds when a cell divides (2, 4, 6, 8 and 10 a.u., to ensure symmetric divisions), cell size increments during cell elongation (3, 4, 5, 6, 7, 8 a.u. per time step) and final length of differentiated cells (75, 80, 85 and 90 a.u.). In all cases we found the following shared features (Table S5): (1) indeterminate growth of Wt roots and determinate growth of *mto2-2* roots; (2) higher free Thr content of *mto2-2* whole roots in comparison with Wt; (3) the deficiency of free Thr in the SCN of *mto2-2* in contrast to Wt roots; (4) a time window where the length of RAM cells in *mto2-2* roots are statistically indistinguishable from the TD cells in Wt roots (unpaired two-tailed Student's *t*-test). As expected, the differences found in these simulations are related to the final root length and total number of cells produced during the simulation. This analysis indicates that the model results are robust in relation to the parameters controlling the cell growth dynamics and thus emerge due to the predicted compartmentalization of free Thr distribution (Table S5). To assess the effects of different expression level of transcripts encoding Thr-related enzymes, we made simulations in which the graded expression was maintained longitudinally but the level of expression was gradually increased or decreased (Fig. S13). We found that the changes did not alter the general outcome of the Wt and *mto2-2* simulations of root growth. These predictions also revealed that decreasing the expression levels to zero caused Wt roots to stop growing, which is consistent with the crucial role of free Thr for growth, and increasing the expression levels restored indeterminate root growth in the *mto2-2* mutant*.* Although it is unclear whether the expression pattern of the Thr-related enzymes changes as roots age, our simulations show that only very drastic alterations will affect root growth in Wt roots, confirming the robustness of the model.

The expression level of *MTO2* and *TSY2* were determined with a position-dependent function derived from a linear regression analysis of the gene expression data reported by Wendrich et al. (2017). This replicates their graded expression in the simulation platform. Similar patterns of gene expression were found in other studies (Denyer et al., 2019; Ryu et al., 2019; Zhang et al., 2019) and in experiments reported here (Fig. S2).

### Modeling Thr synthesis and catabolism

Thr biosynthesis is part of the aspartate-derived amino acid pathway for which a detailed model has been proposed (Curien et al., 2009), although it did not consider the variations that occur in different cells within a tissue. In this study, we aimed to describe Thr metabolism at the multicellular level, adding a layer of complexity not considered previously, and thus we limited the number of enzymatic steps to those directly impacting Thr metabolism. Based on both zone-specific transcriptomic analysis (Brady et al., 2007; Wendrich et al., 2017; Winter et al., 2007) and single cell transcriptomics (Denyer et al., 2019; Ryu et al., 2019; Zhang et al., 2019), *MTO2* is highly expressed in the RAM, and its expression gradually decreases toward the EZ and DZ. This coincides with the *proMTO2:MTO2-GFP* expression pattern (Reyes-Hernández et al., 2019; Fig. S2), indicating that a protein graded distribution is similar to the *MTO2* transcriptional gradient. The expression of *TSY2* has a low level in the RAM and increases gradually towards differentiated root cells and is relatively high in the shoot apex (Brady et al., 2007; Wendrich et al., 2017; Winter et al., 2007). Previously published gene expression data of RAM cells at different distances from the QC (Wendrich et al., 2017) was harnessed to obtain linear functions to describe changes in the expression level of *MTO2* and *TSY2* along the RAM (Fig. 2A), specifically the expression levels and the slopes at which expression changes along the RAM. Then, these linear functions were used in the *in silico* root model to define the expression of each enzyme. The dynamics of Thr biosynthesis are modeled for each cell using an ODE (Eqn 1) that assumes that the activity of the enzymes directly involved in Thr synthesis is proportional to their gene expression level and that the system is at steady state. This assumption is based on the fact that in *A. thaliana* there is a correlation between a transcript and protein abundance (Wendrich et al., 2017) in general, and between aspartate-derived amino acid free content, enzyme activity and the expression level of related genes, in particular (Avraham and Amir, 2005; Clark and Lu, 2015). The ODE consists of production terms calculated with Michaelis-Menten kinetics (assuming a constant supply of substrate, *S*) that considers the individual contribution of two threonine synthase enzymes (MTO2 and TSY2), and a decay term (δ) that represents the usage of Thr for protein synthesis and catabolism. Thus, Thr is modeled as a continuous variable that evolves as a function of MTO2 and TSY2. The equation takes the form:
(1)

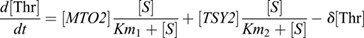
where [*MTO2*] and [*TSY2*] are the concentration of the threonine synthase enzymes catalyzing the reactions, *S* is the substrate (*O*-phosphohomoserine), assumed to be in a constant supply, and *Km_1_* and *Km_2_* are the activity level of the respective enzyme (MTO2 or TSY2) at which production of free Thr is half-maximal. It has been experimentally estimated that in the Wt, the activity of MTO2 is two-orders of magnitude higher than that of TSY2 (Curien et al., 2009). We implemented this in the model for Wt as a difference in *Km* (*Km_2_*≫*Km_1_*). To investigate the behavior of the system we explored a wide range of parameters (1-1000 for *Km* and 1-10 for *δ*, a total of 10,000 simulations). As selection criteria for these production parameters for the *in silico* growing root, we defined that the total free Thr content in *mto2-2* roots has to be higher than that of Wt roots, ∼1.5-fold, as experimentally established (Reyes-Hernández et al., 2019). This simplified model (based only on Thr synthesis) assumes an unlimited pool of precursors for Thr biosynthesis (other key enzymes of the aspartate pathway, i.e. *MTO1* and *MTO3*, do not have a graded expression pattern; Denyer et al., 2019). The model also assumes a constant consumption of free Thr for either catabolism or protein synthesis, a negligible role of protein degradation as a source of free Thr and a relatively low level of S-adenosylmethionine, an allosteric regulator of the threonine synthase enzymes. In relation to the latter, there is no evidence of high methionine or S-adenosylmethionine biosynthesis rate in Wt root apex. However, if this happened to be the case, we would expect an even higher activity of MTO2 and TSY2 enzymes compared with those modeled here, and the predictions of the model may only change in magnitude. Therefore, the generalized predicted pattern of Thr distribution is expected to be conserved and driven by the expression pattern of the modeled enzymes. Thus, this model allows the generation of qualitative predictions regarding the distribution of free Thr along the root.

The ODE for Thr production was also used for the extended model of Thr synthesis and catabolism. As before, the expression of *THA1*, *THA2* and *OMR1* was defined with linear functions derived from a linear regression analysis of gene expression data reported by Wendrich et al. (2017)*.* In this extended model, we also included equations for Gly and Ile derived from Thr catabolism, resulting in the following system of ODEs:
(2)

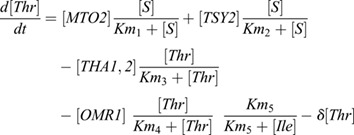

(3)

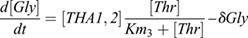

(4)

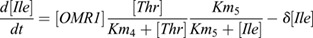


The ODE of Thr production and catabolism (Eqn 2) includes two additional terms to represent the catabolism mediated by THA1/2 and by OMR1. For the latter we included the negative feedback from Ile to OMR1 activity (Xing and Last, 2017). In these equations [*THA1*,*2*] and [*OMR1*] are the concentration of the enzymes mediating Thr catabolism, *Km_3_* and *Km_4_* indicate the level of activity of THA1/2 and OMR1 at which production of Thr-derived Gly and Ile is half-maximal, and *Km_5_* is the level of Ile at which negative regulation of OMR1 is half-maximal. Eqns 3 and 4 include a term of production (via THA1/2 or OMR1, respectively) and of usage (*δ*). As before, we used a wide range of parameters (1-100 for *Km_3_* for THA1/2, 1-100 for *Km_4_* for OMR1 and 1-100 for *Km_5_* for Ile feedback, a total of 125,000 simulations) to explore the free Thr distribution considering both synthesis and catabolism. The ODE models were solved using the R package ‘deSolve’ (Soetaert et al., 2010) and the *in silico* root model was implemented in R (http://cran.R-project.org/) and is available in GitHub (https://github.com/moneralee/A-mutation-in-THREONINE-SYNTHASE-1-uncouples-proliferation-and-transition-domains-of-the-RAM).

### Statistical analyses and figure preparation

Irrespectively of quantitative or qualitative analyses, at least two independent experiments were performed. The number of repetitions and tests used for statistical analysis is indicated in the figure legends and tables. The statistical analysis was performed using SigmaPlot, version 12 (Systat Software) or R-package (http://cran.R-project.org/). A minimal sample size for cell length profile analysis was calculated for an unpaired two-sample *t*-test with a two-tailed alternative hypothesis at a significance level of 0.05 and a test power of 0.9 and was found to be *n*=6.

For figure preparation we used Adobe Photoshop. Red signal was transformed to magenta using the Lookup table of Image J or an option of the Adobe Photoshop CS3 for selective color, adjusting a yellow channel in reds to −100. All CLSM images were single sections without further modifications, except those in Fig. S6. In this figure, images were deconvolved using the theoretical PSF generator ‘Diffraction PSF 3D plugin’ (https://www.optinav.info/Diffraction-PSF-3D.htm) and the Parallel Iterative Deconvolution plugin of Image J (https://sites.google.com/site/piotrwendykier/software/deconvolution/paralleliterativedeconvolution) followed by the automatic mode of PureDenoise plugin of Image J (http://bigwww.epfl.ch/algorithms/denoise/). Cell length profile graphs were generated by Python libraries matplotlib (Hunter, 2007) and seaborn (Waskom, 2021). Figs 2,7 and Figs S3,S11,S12 were generated with R, with the exception of Fig. 2B, Figs S3B and S12B, which were generated with the Python library ‘Violin SuperPlots’ (Kenny and Schoen, 2021).

## Supplementary Material

10.1242/develop.200899_sup1 Supplementary information
