# Supplementary material for: A mutation in THREONINE SYNTHASE 1 uncouples proliferation and transition domains of the root apical meristem: experimental evidence and in silico proposed mechanism
Source: Development. 2022 Nov 9;149(21):dev200899. doi: 10.1242/dev.200899 (PMC9796171; doi:10.1242/dev.200899)
Supplement: Supplementary information [file develop-149-200899-s1.pdf]

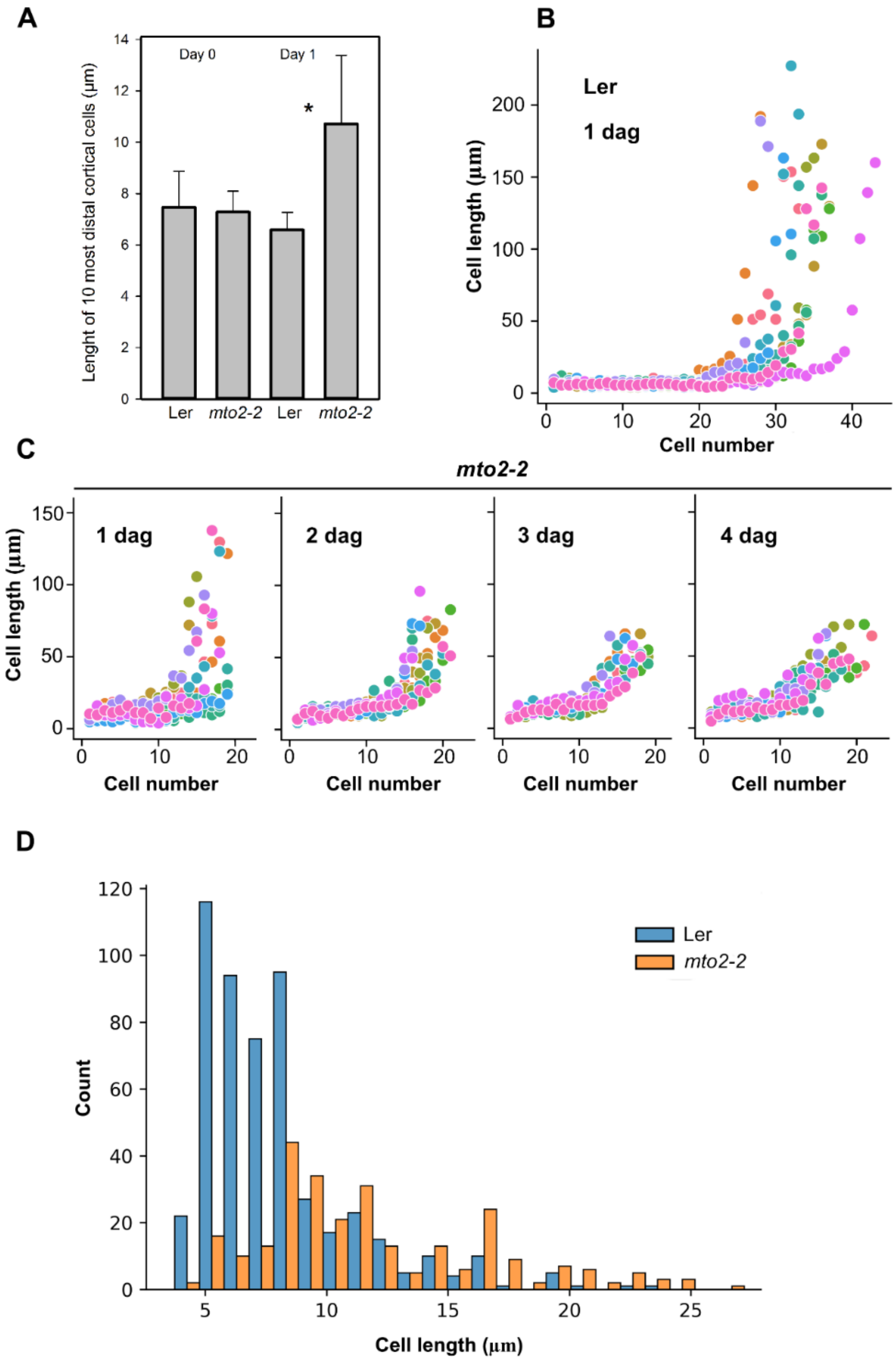

Fig. S1. Differences in Cell Length Distribution of the RAM cells in Wt and the *mto2-2* mutant. (A) Average length of 10 distal cortex cells in roots of seedlings at 0 and 1 dag. Asterisk indicates statistical difference determined by the Student's *t*-test, $P<0.001$, n = 12. Mean±SD. (B) Cell length profile in roots of 1 dag Wt seedlings. (C) Cell length profile in roots of 1-4 dag *mto2- 2* seedlings. (B) and (C) show the data of an experiment independent of that shown in Figure 1 of the main text; n=12; cortex cells were measured. (D) Cortical cell length distribution of the RAM cells in Wt and the *mto2-2* mutant in 1 dag seedlings. The length of the RAM was detected arbitrarily up to a cell that started rapid elongation. Combined data of two independent experiments; total number of measured cells was 299 for *mto2-2* and 522 for Wt; n = 19 roots.

Development • Supplementary information

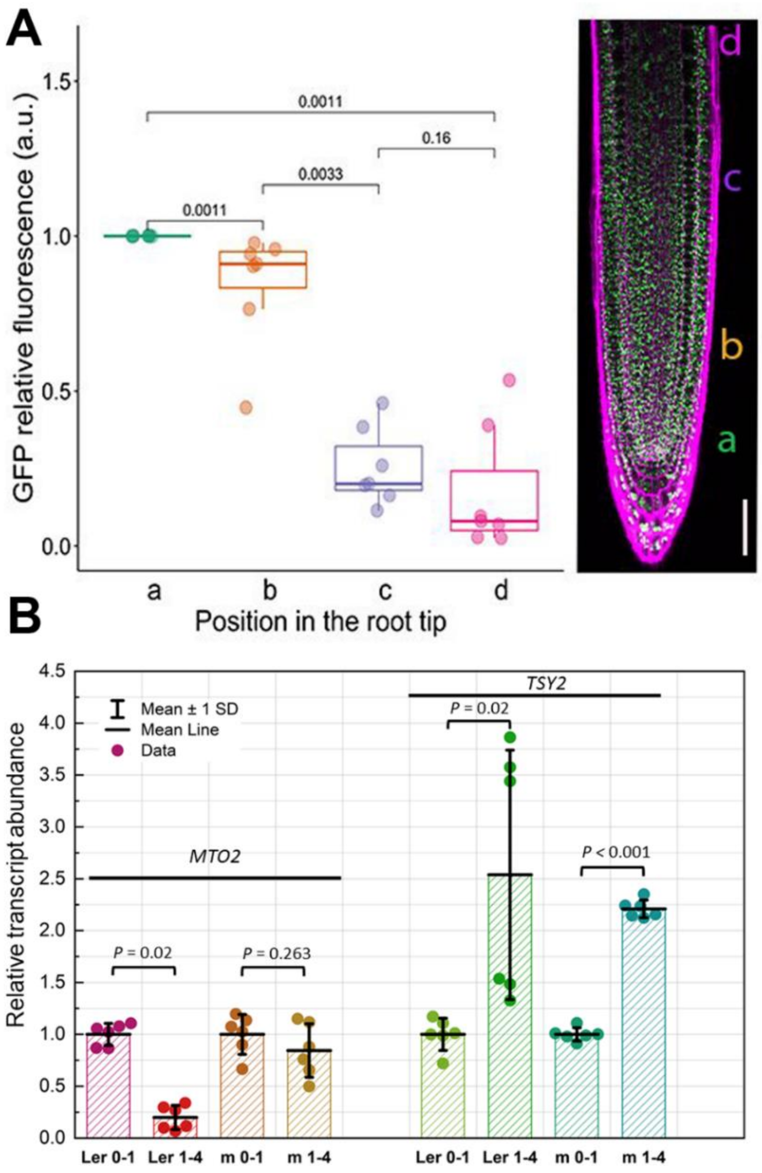

Fig. S2. ***MTO2* and *TSY2* expression gradient along the primary root in Wt and the *mto2-2* mutant**. (A) Relative GFP intensity in *pMTO2:MTO2-GFP* root tip was measured in the cortical layer, starting from the ground tissue initial (stem) cells (position a), cell in the middle portion of the proliferation domain of the root apical meristem (position b), cell in middle portion of the transition domain of the root apical meristem (position c), and the first cell of the elongation zone (position d), *n*=7. Statistical significance between samples was evaluated using two-tailed Wilcoxon test. (B) Relative transcript abundance of *MTO2* and *TSY2* within the same genotype of Ler (Wt) and the *mto2-2* mutant (m) in the root portions 0-1 and 1-4 mm from the root tip. Analysis was performed with two biological and three technical replicates. Student's *t*-test was used here and below. In addition, the comparison of *MTO2* transcript abundance in the mutant versus Wt showed that in the *mto2-2* it was 57% and 302% of that in Wt (for 0-1 and 1-4 mm portions, with $P<0.001$ and $P = 0.004$, respectively). For *TSY2,* the abundance was 303 and 302% in the mutant compared to Wt (for 0-1 and 1-4 mm portions, with $P = 0.002$ in both cases).

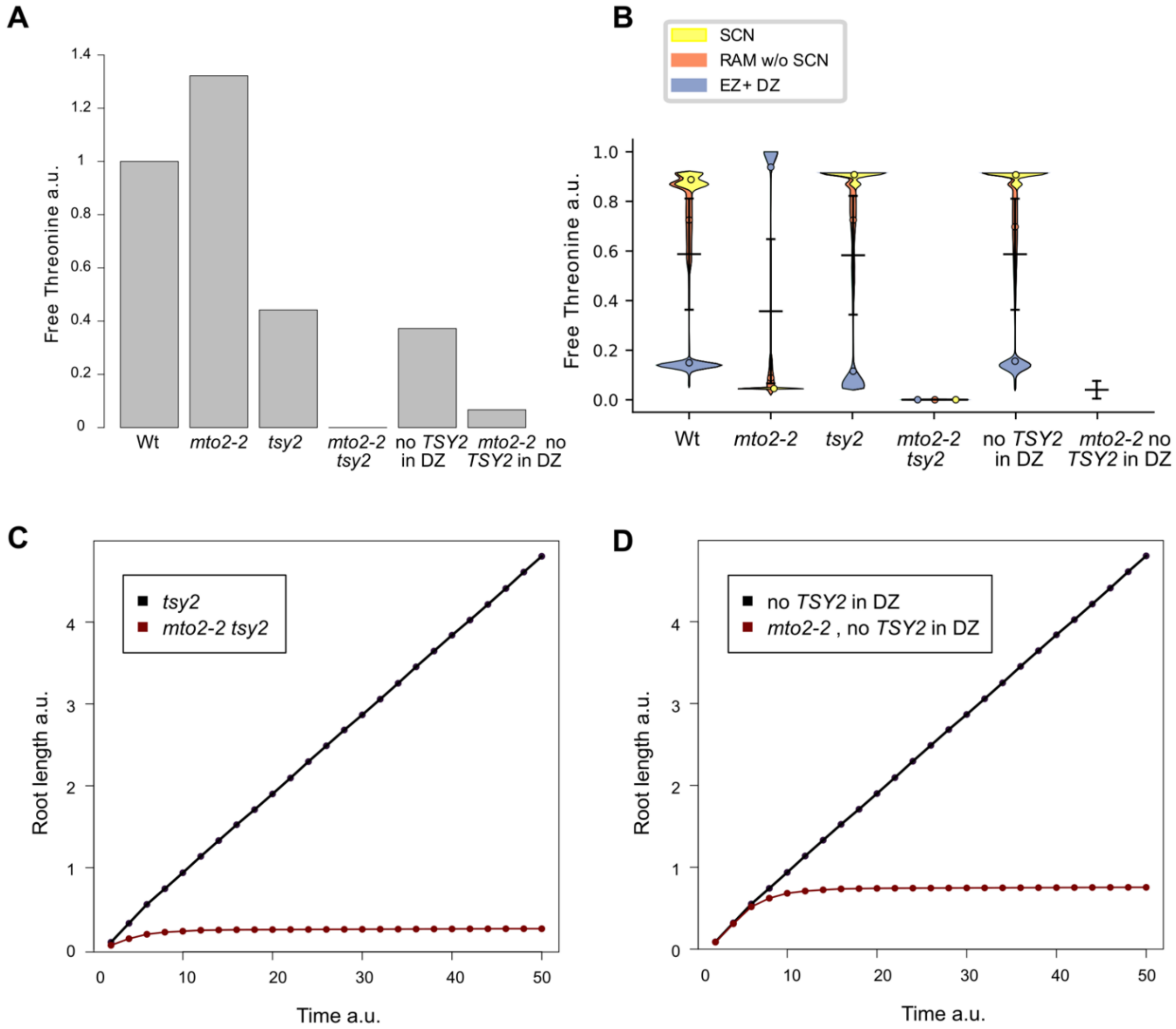

Fig. S3. ***In silico*** **model of free Thr distribution in different compartments in roots of Wt and mutants affected in threonine synthesis and simulation of root growth pattern** (A) Predicted total free Thr levels of Wt, *mto2-2*, *tsy2*, *mto2-2 tsy2 in silico* roots, and a hypothetical mutant with no *TSY2* expression specifically in the DZ in Wt (no *TSY2* in DZ) and *mto2-2* (*mto2-2* no *TSY2* in DZ) background (at time = 5 a.u., see Figure 1C). An increase in free Thr content in whole *in silico mto2-2* roots is due to the putative compensation by TSY2 activity, as no free Thr in *mto2-2 tsy2* should be present according to the simulation. Particularly, TSY2 activity in the DZ drives the increase in free Thr levels in *mto2-2* roots because a hypothetical case with no *TSY2* expression in the DZ in the *mto2-2* background results in diminished free Thr levels. (B) Violin plots of the distribution of free Thr in the *in silico* roots of Wt and mutants as in (A). The colors indicate different subsets of cells within the roots: SCN, RAM (without SCN), and EZ+DZ. Notice that despite the higher free Thr levels in *mto2-2* (A), the SCN cells have marginal levels of free Thr. (C) Root growth curves of *in silico* roots in the *tsy2* mutant and double *mto2-2 tsy2* mutant. The model predicts that in the double mutant, embryonic radicle cells at the initial condition only elongate and no new cell production is possible. (D) Root growth curves of *in silico* roots mutant in a hypothetical case with no *TSY2* expression in the DZ.

Development • Supplementary information

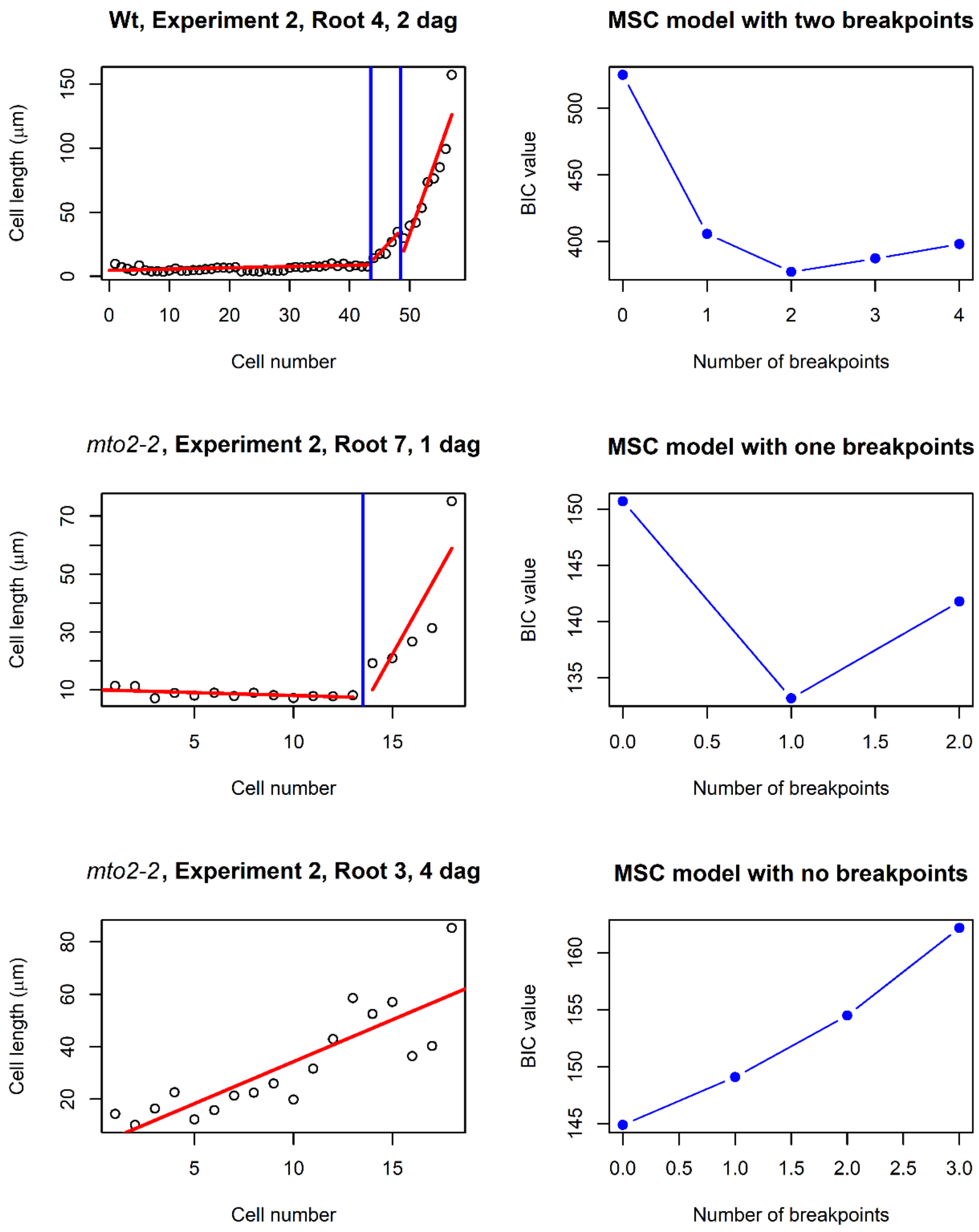

Fig. S4. Estimation of the number of breakpoints and their position by the MSC Approach. Examples of cell length profiles in concrete roots are given. The lowest value of Bayesian Information Criterion (BIC) corresponds to the most parsimonious model of the number of breakpoints within a cell file. Vertical lines show position of a breakpoints estimated by an MSC approach.

Development • Supplementary information

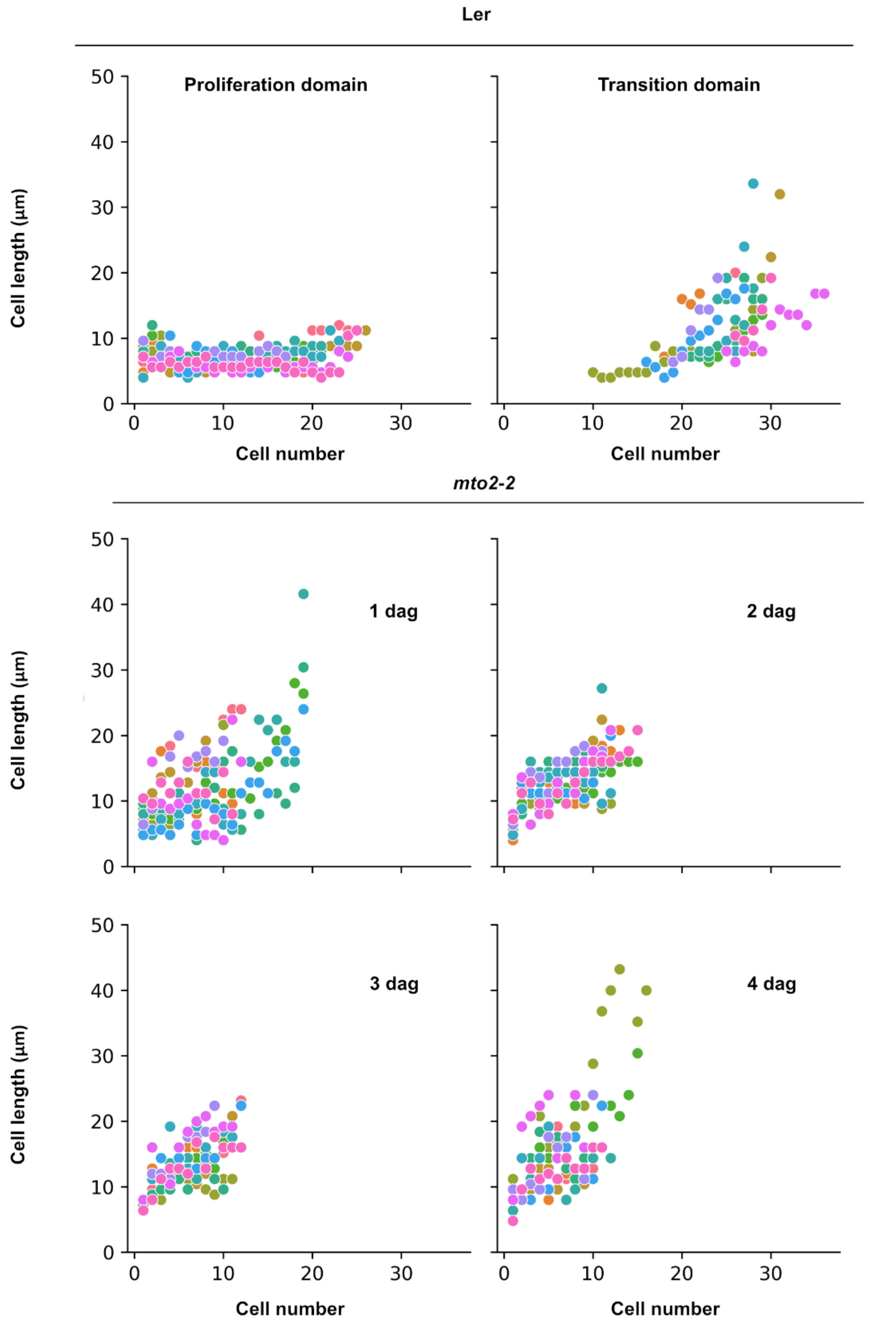

Fig. S5. Proliferation and transition domains of Wt and the RAM in the *mto2-2* mutant. Cell length profile in roots of Wt and *mto2-2* seedlings within the proliferation and transition domain of Wt as identified by the MSC approach and within the RAM of the *mto 2-2* mutants identified by the MSC approach. The data are similar to those in the Figure 3 of the main text, but for an independent experiment (*n*=12). Cortical cell lengths for an individual root are marked with the same color.

Development • Supplementary information

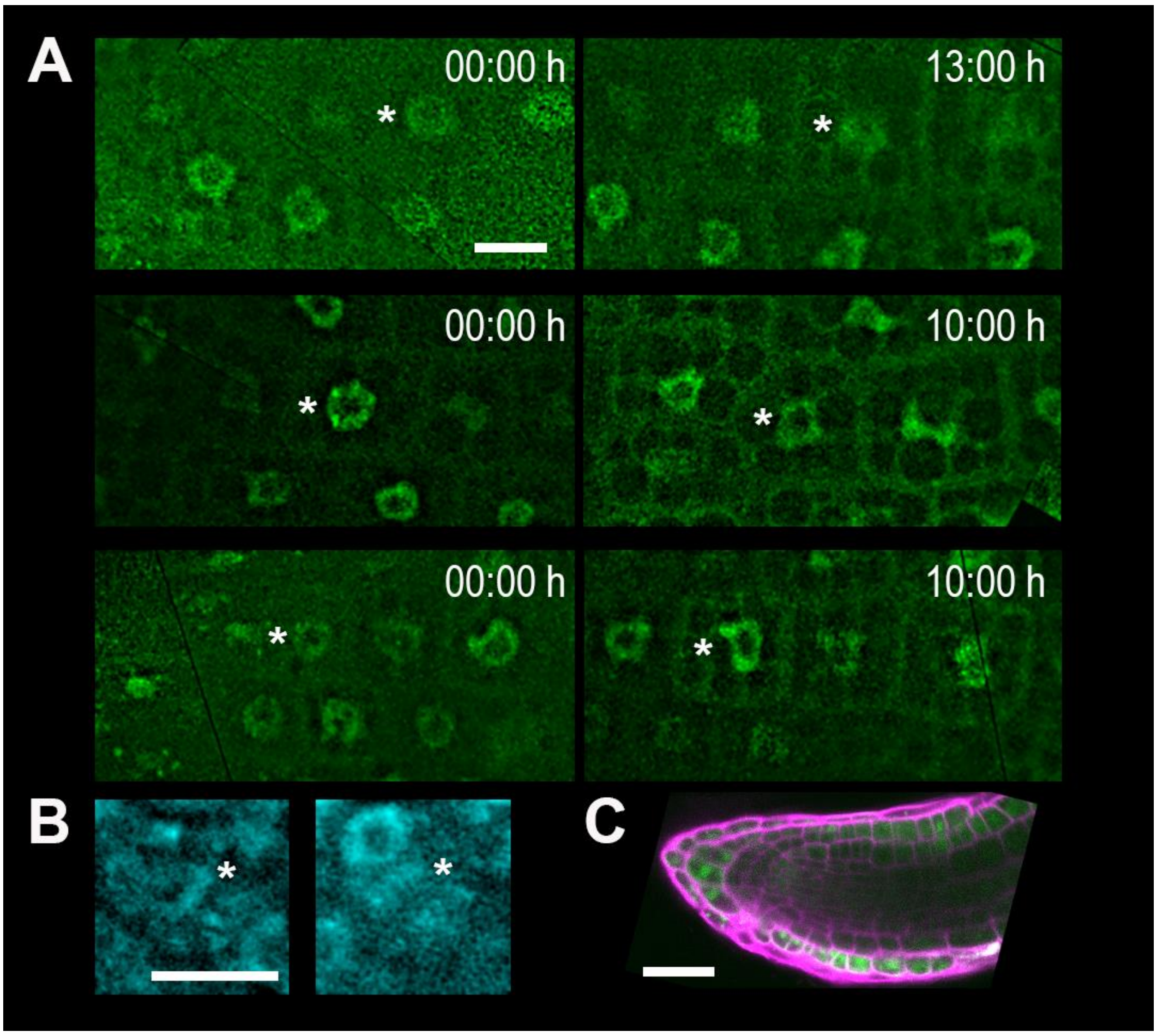

Fig. S6. Analysis of *pAtPCNA1::AtPCNA1-sGFP* expression and mitoses in the *mto2-2 and* the endoreduplication marker, *pCCS52A1::CCS52A1-GFP*. (A) Time-lapse analysis of cells expressing *pAtPCNA1::AtPCNA1-sGFP* (proliferating cell nuclear antigen-sGFP fusion) in the background of the *mto2-2* during a period of 3 to 4 dag. The RAM cells in three different roots are shown in which speckle pattern of *AtPCNA1* (reporting late S-phase) was detected in cells at time 0 and was maintained till the time indicated in right panels. The same cell is marked with asterisk. Note that during the time indicated, cells increased their size. Different roots are maximum intensity projections, from 2 to 8 optical sections, 1 µm of thickness per section; *n* = 6. (B) Rare mitotic figures in the RAM of the *mto2-2* 4 dag seedlings stained with DAPI; metaphase (left image, asterisk) and telophase (right image, asterisk); single optical sections; *n*=12. Due to dense cytoplasm in the mutant, it was not possible to estimate mitotic index in the RAM. Bar: 15 µm. (C) The expression of the endoreduplication marker, *pCCS52A1::CCS52A1-GFP,* in the *mto2-2* seedling of at 2 DAG, *n*=6. Bar: 50 µm.

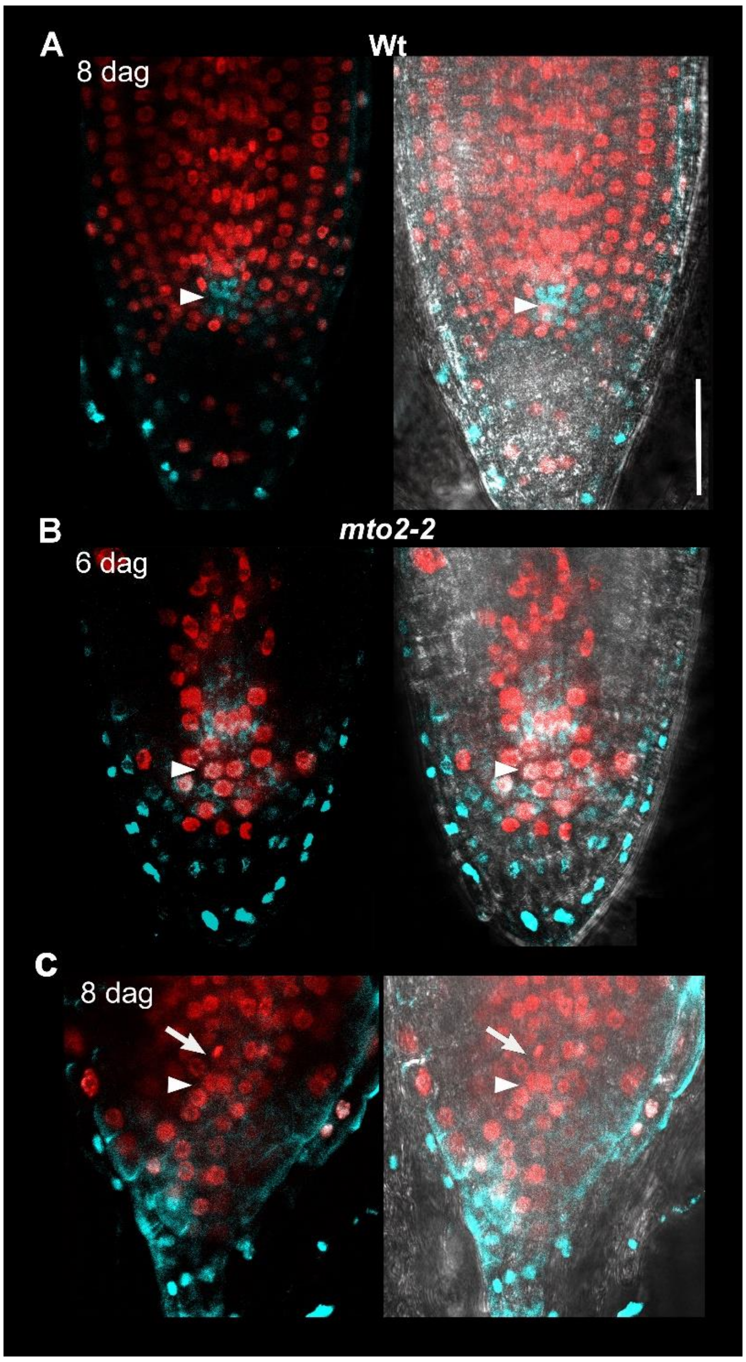

Fig. S7. The *mto2-2* QC cells undergo DNA synthesis during root apical meristem exhaustion. (A) Wild type Ler. (B) and (C) The *mto2-2* seedlings at 5 and 7 dag were transferred to plates with fresh growth medium supplemented with EdU for 24 h. The indicated age refers to the time at the end of the experiment. Arrowhead indicates the position of the QC; arrow shows a provascular stem cell in metaphase. Single optical sections are shown; n=15 (Wt), n=14 (*mto2-2*, 6dag), n=27 (*mto2-2*, 8dag); 3 independent experiments. Bar: 50 µm.

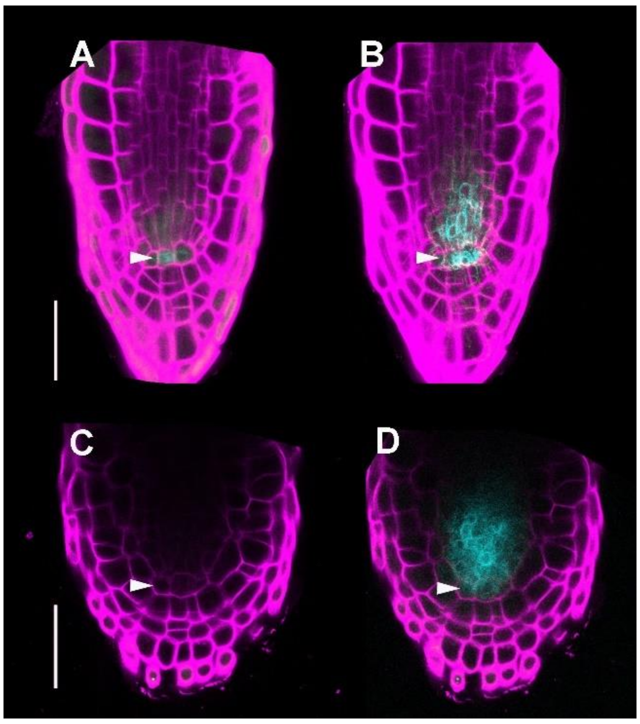

Fig. S8. Methods of analysis of fluorescent signals in the *mto2-2* root tip.
Due to denser cytoplasm and cell expansion during the *mto2-2* root apical meristem exhaustion, CLSM is insufficient to detect internal fluorescent signal. All panels show *mto2-2; proPLT1:CFP* root tip stained with propidium iodide (red channel pseudo-colored to magenta). (A) and (C) show images obtained with CLSM for both red and cyan channels; note weak signal of cyan. (B) and (D) show the same roots as on (A) and (C) in which the CLSM-visualized red channel was merged with cyan channel visualized with two-photon microscopy. Note that cyan fluorescent signal is much stronger when two-photon microscopy is used. (A) and (B) show 7 dag and (C) and (D) 8 dag seedling roots (n = 7). Arrowheads show the QC position. Scale bar = 50 μm.

Development • Supplementary information

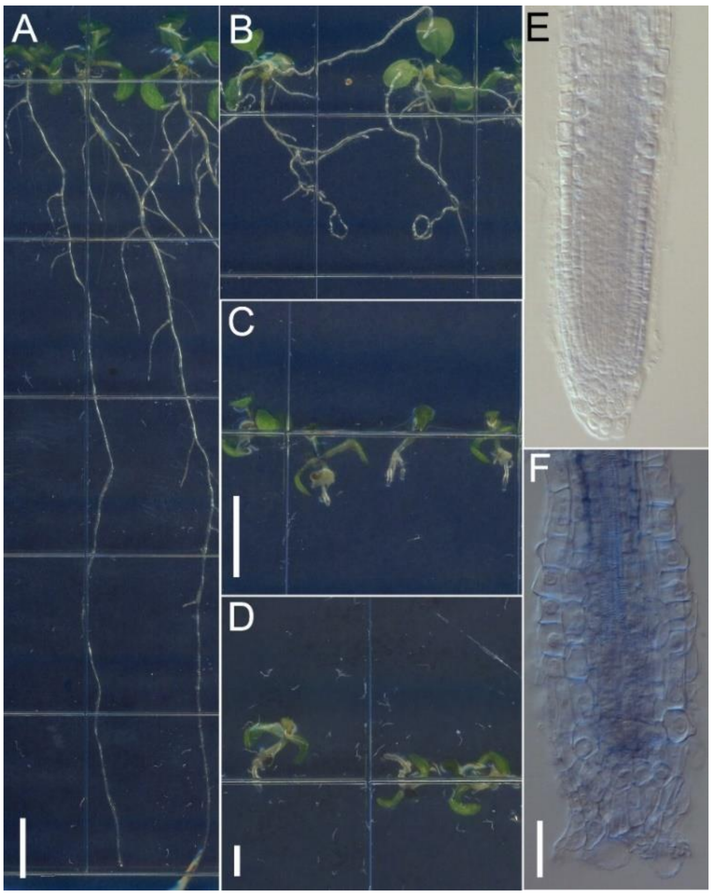

Fig. S9. inhibitor of auxin synthesis, kynurenin, does not affect root apical meristem exhaustion. Young 2 dag Wt (A-B) and *mto2-2* (C-D) seedlings were transferred to fresh growth medium supplemented with DMSO (A) and (C) or 4 μM kynurenine (B) and (D) and grown for additional nine days. (E) and (F) Root apical meristem in 11 dag Ler and in *mto2-2* after kynurenine treatment. Two independent experiments were performed, *n* = 10-40 seedlings. Bar = 10 mm (A) to (E) and 50 μm in (E) and (F).

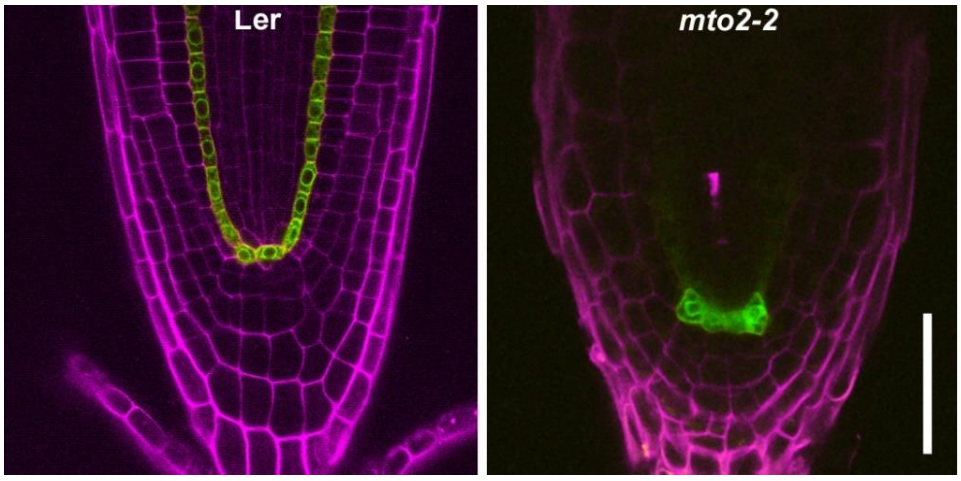

Fig. S10. Expression of *proSCR:GFP* in Wt and *mto2-2* plants. Expression of the marker was analyzed with two-photon microscopy in 9 dag plants. Bar = 50 μm.

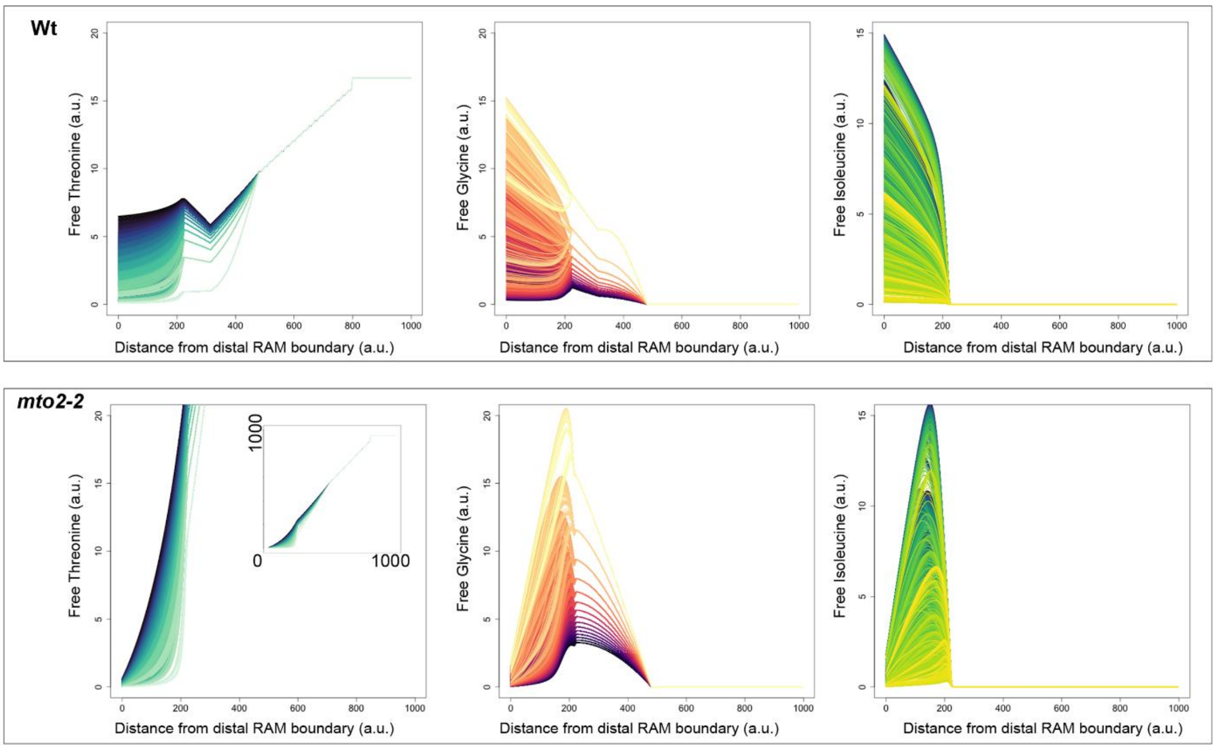

Fig. S11. Free Thr, free Gly, and free Ile distribution for Wt and *mto2-2* roots derived from the extended model considering both synthesis and catabolism. The plots show the distribution predicted by the 125,000 parameter sets used to solve the ODE of the model that combines Thr synthesis and catabolism in the root considering the role of *MTO2, TSY2, THA1, THA*2, and *OMR1*. For *mto*2-2, the inset shows the extent of the increase in the levels of free Thr (notice the different scales of the y axes).

Development • Supplementary information

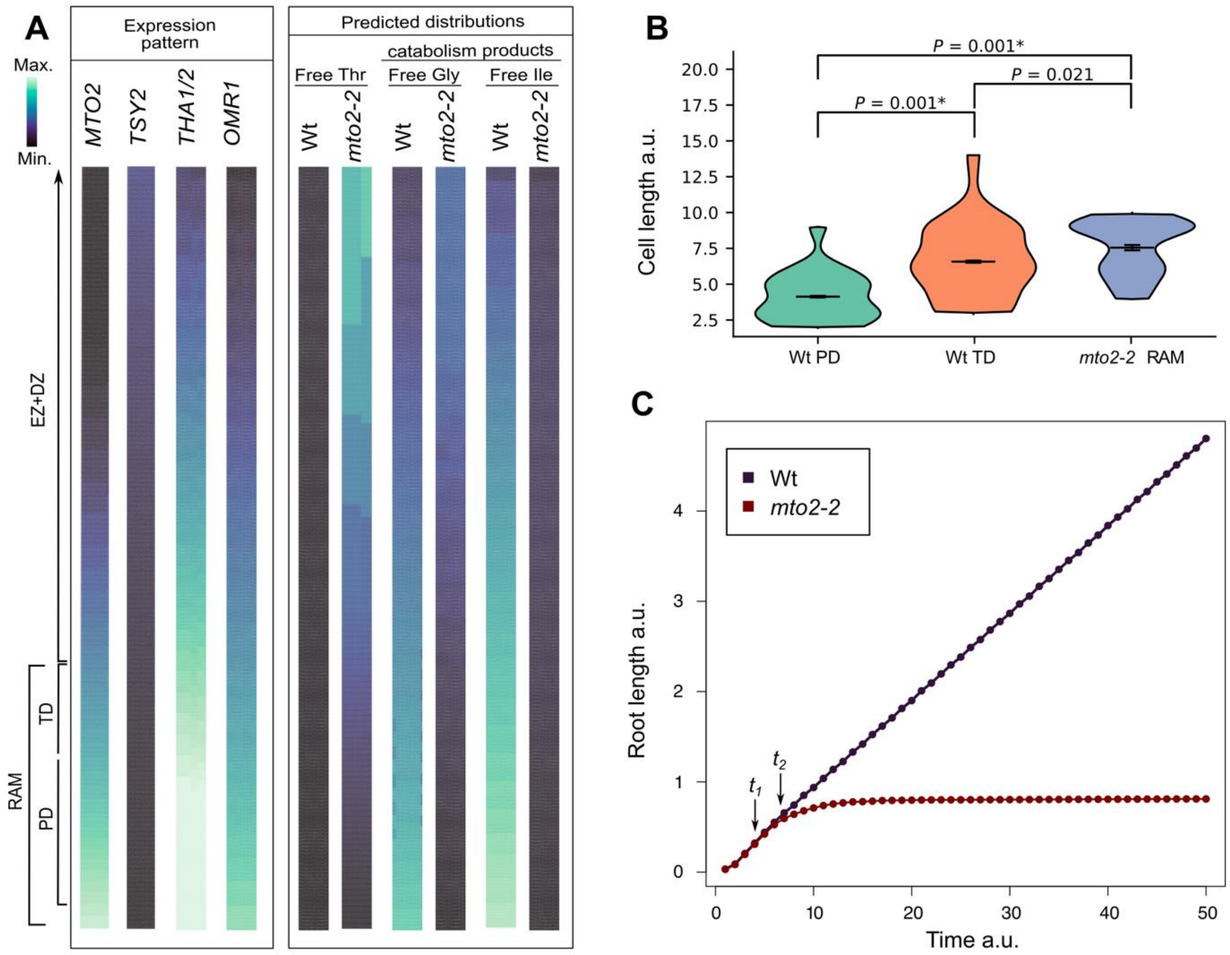

Fig. S12. Mathematical model of Thr synthesis and catabolism in the root tip and prediction of root cell length profile in wild type and the *mto2-2* mutant. (A) Expression patterns along the root of enzymes directly involved in Thr biosynthesis, *MTO2* and *TSY2*, and Thr catabolism, *THA1/2* and *OMR1*, as defined by linear functions based on expression data (see Materials and Methods). Prediction of the distribution of free Thr and Thr-catabolism products (Gly and Ile) in Wt and the *mto2-2* roots. While free Thr levels are rather low in the Wt root apex, Thr-catabolic products are enriched in the RAM. (B) Cell length distribution in the *in silico* modelled roots at a time between t_1 and t_2 shown in (C) when the RAM is still present, length of the RAM cells in the *mto2-2* mutant (184 cells) is similar to that of the TD cells in Wt (78 cells), but not to that in the PD (201 cells); *P* was calculated with two-tailed Student's *t*-test. (C) Root growth curves of Wt and *mto2-2 in silico* roots, the former grows indeterminately while the latter grows for a limited period of time.

Development • Supplementary information

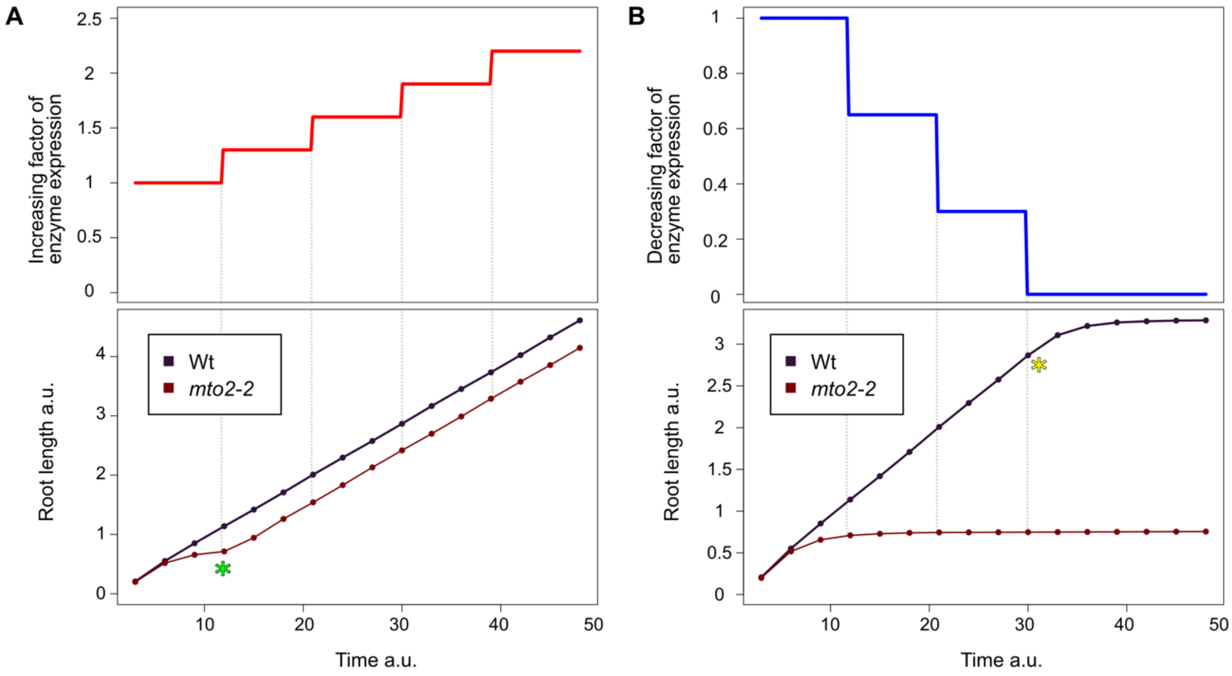

Fig. S13. Robustness of the *in silico* root growth model to changes in the expression level of *MTO2* and *TSY2*. We simulated changes in expression levels by multiplying the variables related to MTO2 and TSY2 by a factor that results in an increase or a decrease expression, respectively (top panels in A and B). (A) Increasing factor of enzyme expression does not affect the growth of Wt *in silico* roots (bottom panel), while it can restore root growth of *mto2-2* given an increased activity of TSY2 in the RAM (green asterisk marks the point where *mto2-2* root growth is rescued). (B) Decreasing factor of enzyme expression (top panel) results in no alterations in Wt and the *mto2-2* mutant in *silico* root growth (bottom panel), until expression is zero (decreasing factor = 0) when Wt roots stop growing (at a time indicated by yellow asterisk). This root growth arrest is caused by the lack of MTO2 activity.

Table S1. Characteristics of the proliferation and transition domains in the root apical meristem of Wt and the *mto2-2* mutant identified by the MSC algorithm.

	Ler				*mto2-2*			
Time (dag)	1	2	3	4	1	2	3	4
Number of cells in the PD	19.8 ± 4.1	33.3 ± 5.9	38.7 ± 8.7	40.4 ± 11.4	0.6 ± 1.9 ***	2.2 ± 3.4***	2.1 ± 3.6***	2.1 ± 3.2***
Number of cells in the TD	8.1 ± 4.1	11.4 ± 4.7	17.9 ± 7.3	10.7 ± 3.7	12.2 ± 4.3**	9.4 ± 3.9	8.6 ± 3.4**	8.2 ± 4.3
Length of the PD (µm)	146.4 ± 33.8	216.8 ± 57.8	261.1 ± 68.6	274.6 ± 73.5	8.0 ± 24.6***	29.2 ± 45.8***	31.4 ± 54.7***	35.8 ± 61.4***
Length of the TD (µm)	99.9 ± 28.4	136.6 ± 34.7	204.1 ± 57.8	149.2 ± 80.7	151.6 ± 55.4**	124.2 ± 46.9	128.3 ± 49.9*	155.8 ± 109.4
Cell length in the PD (µm)[a]	7.4 ± 2.0 (19/376)	6.5 ± 1.9 (7/233)	6.7 ± 1.9 (7/271)	6.8 ± 1.9 (7/283)	12.6 ± 4.7 (2/12) ***	13.6 ± 3.7 (6/41) ***	14.9 ± 4.5 (6/40) ***	17.0 ± 6.9 (6/40) ***
Cell length in the TD (µm)[a]	12.4 ± 7.4 (19/147)	12.0± 4.5 (7/80)	11.4 ± 4.5 (7/125)	13.9 ± 5.5 (7/75)	12.5 ± 7.1 (19/231)	13.3 ± 4.1 (19/178) **	15.0 ± 4.4 (19/163) ***	19.9 ± 12.6 (17/143) ***
n	19	7	7	7	19	19	19	19

Data are means ± SD. [a]The first number in the parenthesis is the number of roots in which the respected domain is detected by the MSC algorithm; the second number indicates total number of cortical cells in the respected domain of these roots. Statistical differences at **P* < 0.05. ***P* < 0.01. ****P* < 0.001 were determined by the Mann-Whitney test.

Table S2. Number of transitions (breakpoints) determined by the Bayesian Information Criterion (BIC) when cell length profiles are analyzed with the MSC approach. Combined data of two independent experiments.

Genotype	Number of roots with			
**Time (dag)**	**No transitions**	**One transition**	**Two transitions**	**Total**
mto2-2				
**1 dag**	1 (5 %)	14 (74 %)	4 (21 %)	19
**2 dag**	0 (0 %)	13 (68 %)	6 (32 %)	19
**3 dag**	0 (0 %)	13 (68 %)	6 (32 %)	19
**4 dag**	3 (15.8 %)	10 (52.6 %)	6 (31.6 %)	19
**Total**	**4 (5%)**	**50 (66%)**	**22 (29 %)**	**76**

Development • Supplementary information

Table S3. Determination of the *h* value for MSC modeling of cell length profile in each root

Genotype	Experiment number	dag	Root number	Number of cells in the profile	*f value*	*h* value
mto2-2	1	1	1	18	0.24	4
			2	19		5
			3	14*		3
			4	15		4
			5	19		5
			6	19		5
			7	19		5
			8	18		4
			9	19		5
			10	16		4
			11	18		4
			12	17		4
		2	1	18		4
			2	20		5
			3	19		5
			4	18		4
			5	21		5
			6	16		4
			7	16		4
			8	20		5
			9	17		4
			10	16		4
			11	17		4
			12	21		5
		3	1	17		4
			2	16		4
			3	18		4
			4	19		5
			5	19		5
			6	19		5
			7	15		4
			8	18		4
			9	17		4
			10	14		3
			11	17		4
			12	18		4
		4	1	22		5
			2	15		4
			3	16		4
			4	19		5

Development • Supplementary information

			5	21		5
			6	17		4
			7	18		4
			8	15		4
			9	16		4
			10	16		4
			11	15		4
			12	19		5
**Wt**	1	1	1	33	0.12	4
			2	28*		3
			3	37		4
			4	35		4
			5	37		4
			6	36		4
			7	33		4
			8	33		4
			9	32		4
			10	29		3
			11	43		5
			12	36		4
mto2-2	2	1	1	17	0.24	4
			2	19		5
			3	18		4
			4	21		5
			5	19		5
			6	15		4
			7	18		4
		2	1	16		4
			2	18		4
			3	15		4
			4	17		4
			5	16		4
			6	17		4
			7	19		5
		3	1	19		5
			2	16		4
			3	13*		3
			4	21		5
			5	16		4
			6	20		5
			7	16		4
		4	1	17		4
			2	15		4
			3	18		4
			4	17		4
			5	16		4

			6	17		4
			7	16		4
Wt	2	1	1	27	0.13	4
			2	29		4
			3	31		4
			4	35		5
			5	38		5
			6	25*		3
			7	34		4
		2	1	58		8
			2	45		6
			3	57		7
			4	57		7
			5	65		8
			6	48		6
			7	58		8
		3	1	68		9
			2	67		9
			3	77		10
			4	68		9
			5	62		8
			6	78		10
			7	63		8
		4	1	72		9
			2	62		8
			3	74		10
			4	71		9
			5	65		8
			6	42		5
			7	60		8

*The smallest cell length profile of the genotype in a single experiment.

Table S4. Primers used for qRT-PCR reactions

Gene	Primers		
EF-1α	Fw	AGGCTGGTATCTCCAAGGATGG	
	Rev	TGGCATCCATCTTGTTACAACAGC	Czechowski *et al*., 2005
UBQ10	Fw	TGACAACGTGAAGGCCAAGAT CC	
	Rev	ATACCTCCACGCAGACGCAACAC	Czechowski *et al*., 2005
MTO2	Fw	GCCTCTGTATCGTCGTTAAACG	
	Rev	GCGGTACAGGAGATGACGAC	This study
TSY2	Fw	CTCCGCCACCTACTTTCCTTC	
	Rev	GGGTTTCTGTGGAGTCTGTGG	This study

Development • Supplementary information

Table S5. Sensibility analysis of root growth parameters used for *in silico* root modelling of Thr biosynthesis in Wt and the *mto2-2* mutant. Simulations were performed varying the parameters controlling the size at which cells divide in the RAM (Dv.), how fast cells elongate (El.) and size at which cells differentiate (Df.). In all cases the simulations obtained the following results (Criteria):

1. Indeterminate growth for Wt roots and determinate growth for *mto2-2* roots are maintained.
2. Free Thr level ([Thr]) in cells of Wt is distributed in the following gradient: [Thr] in SCN>[Thr] in RAM (without SCN)>[Thr] in EZ+DZ. In the *mto2-2* mutant, the distribution of [Thr] is the following: [Thr] in EZ+DZ>[Thr] in RAM (without SCN)>[Thr] in SCN cells.
3. Total free Thr in *mto2-2* roots is greater than that in Wt full roots.
4. Lengths of cells in the TD of Wt is statistically the same as in the RAM cells of the *mto2-2* mutant (two-tailed Student *t*-test).

Parameters			Criteria				Final Root Length		Final Cell number	
**Dv.**	**El.**	**Df.**	**1**	**2**	**3**	**4**	**Wt**	***mto2-2***	**Wt**	***mto2-2***
2	3	75	✓	✓	✓	✓	9500	3155	9009	2338
2	3	80	✓	✓	✓	✓	9500	3180	9009	2338
2	3	85	✓	✓	✓	✓	9500	3204	9009	2338
2	3	90	✓	✓	✓	✓	9500	3213	9009	2338
2	4	75	✓	✓	✓	✓	10777	3529	9009	2338
2	4	80	✓	✓	✓	✓	10777	3565	9009	2338
2	4	85	✓	✓	✓	✓	10777	3585	9009	2338
2	4	90	✓	✓	✓	✓	10777	3601	9009	2338
2	5	75	✓	✓	✓	✓	12063	3880	9009	2338
2	5	80	✓	✓	✓	✓	12063	3909	9009	2338
2	5	85	✓	✓	✓	✓	12063	3934	9009	2338
2	5	90	✓	✓	✓	✓	12063	3954	9009	2338
2	6	75	✓	✓	✓	✓	13139	4199	9009	2338
2	6	80	✓	✓	✓	✓	13139	4239	9009	2338

2	6	85	✓	✓	✓	✓	13139	4263	9009	2338
2	6	90	✓	✓	✓	✓	13139	4287	9009	2338
2	7	75	✓	✓	✓	✓	14154	4512	9009	2338
2	7	80	✓	✓	✓	✓	14154	4551	9009	2338
2	7	85	✓	✓	✓	✓	14154	4572	9009	2338
2	7	90	✓	✓	✓	✓	14154	4600	9009	2338
2	8	75	✓	✓	✓	✓	15309	4785	9009	2338
2	8	80	✓	✓	✓	✓	15309	4837	9009	2338
2	8	85	✓	✓	✓	✓	15309	4877	9009	2338
2	8	90	✓	✓	✓	✓	15309	4893	9009	2338
4	3	75	✓	✓	✓	✓	7333	2631	5683	1491
4	3	80	✓	✓	✓	✓	7333	2656	5683	1491
4	3	85	✓	✓	✓	✓	7333	2680	5683	1491
4	3	90	✓	✓	✓	✓	7333	2689	5683	1491
4	4	75	✓	✓	✓	✓	8310	2916	5683	1491
4	4	80	✓	✓	✓	✓	8310	2952	5683	1491
4	4	85	✓	✓	✓	✓	8310	2972	5683	1491
4	4	90	✓	✓	✓	✓	8310	2988	5683	1491
4	5	75	✓	✓	✓	✓	9222	3194	5683	1491
4	5	80	✓	✓	✓	✓	9222	3223	5683	1491
4	5	85	✓	✓	✓	✓	9222	3248	5683	1491
4	5	90	✓	✓	✓	✓	9222	3268	5683	1491
4	6	75	✓	✓	✓	✓	10150	3426	5683	1491
4	6	80	✓	✓	✓	✓	10150	3466	5683	1491
4	6	85	✓	✓	✓	✓	10150	3490	5683	1491
4	6	90	✓	✓	✓	✓	10150	3514	5683	1491
4	7	75	✓	✓	✓	✓	10881	3637	5683	1491

4	7	80	✓	✓	✓	✓	10881	3683	5683	1491
4	7	85	✓	✓	✓	✓	10881	3697	5683	1491
4	7	90	✓	✓	✓	✓	10881	3725	5683	1491
4	8	75	✓	✓	✓	✓	11734	3856	5683	1491
4	8	80	✓	✓	✓	✓	11734	3908	5683	1491
4	8	85	✓	✓	✓	✓	11734	3948	5683	1491
4	8	90	✓	✓	✓	✓	11734	3964	5683	1491
6	3	75	✓	✓	✓	✓	5515	2314	3414	1009
6	3	80	✓	✓	✓	✓	5515	2337	3414	1009
6	3	85	✓	✓	✓	✓	5515	2355	3414	1009
6	3	90	✓	✓	✓	✓	5515	2367	3414	1009
6	4	75	✓	✓	✓	✓	6287	2553	3414	1009
6	4	80	✓	✓	✓	✓	6287	2587	3414	1009
6	4	85	✓	✓	✓	✓	6287	2603	3414	1009
6	4	90	✓	✓	✓	✓	6287	2619	3414	1009
6	5	75	✓	✓	✓	✓	6976	2777	3414	1009
6	5	80	✓	✓	✓	✓	6976	2809	3414	1009
6	5	85	✓	✓	✓	✓	6976	2834	3414	1009
6	5	90	✓	✓	✓	✓	6976	2854	3414	1009
6	6	75	✓	✓	✓	✓	7633	2965	3414	1009
6	6	80	✓	✓	✓	✓	7633	3003	3414	1009
6	6	85	✓	✓	✓	✓	7633	3033	3414	1009
6	6	90	✓	✓	✓	✓	7633	3057	3414	1009
6	7	75	✓	✓	✓	✓	8158	3148	3414	1009
6	7	80	✓	✓	✓	✓	8158	3187	3414	1009
6	7	85	✓	✓	✓	✓	8158	3215	3414	1009
6	7	90	✓	✓	✓	✓	8158	3229	3414	1009

6	8	75	✓	✓	✓	✓	8779	3317	3414	1009
6	8	80	✓	✓	✓	✓	8779	3367	3414	1009
6	8	85	✓	✓	✓	✓	8779	3399	3414	1009
6	8	90	✓	✓	✓	✓	8779	3415	3414	1009
8	3	75	✓	✓	✓	✓	4481	2068	2371	768
8	3	80	✓	✓	✓	✓	4481	2086	2371	768
8	3	85	✓	✓	✓	✓	4481	2107	2371	768
8	3	90	✓	✓	✓	✓	4481	2123	2371	768
8	4	75	✓	✓	✓	✓	5093	2282	2371	768
8	4	80	✓	✓	✓	✓	5093	2310	2371	768
8	4	85	✓	✓	✓	✓	5093	2326	2371	768
8	4	90	✓	✓	✓	✓	5093	2346	2371	768
8	5	75	✓	✓	✓	✓	5630	2460	2371	768
8	5	80	✓	✓	✓	✓	5630	2486	2371	768
8	5	85	✓	✓	✓	✓	5630	2506	2371	768
8	5	90	✓	✓	✓	✓	5630	2526	2371	768
8	6	75	✓	✓	✓	✓	6125	2629	2371	768
8	6	80	✓	✓	✓	✓	6125	2654	2371	768
8	6	85	✓	✓	✓	✓	6125	2684	2371	768
8	6	90	✓	✓	✓	✓	6125	2708	2371	768
8	7	75	✓	✓	✓	✓	6589	2790	2371	768
8	7	80	✓	✓	✓	✓	6589	2825	2371	768
8	7	85	✓	✓	✓	✓	6589	2853	2371	768
8	7	90	✓	✓	✓	✓	6589	2867	2371	768
8	8	75	✓	✓	✓	✓	7009	2925	2371	768
8	8	80	✓	✓	✓	✓	7009	2958	2371	768
8	8	85	✓	✓	✓	✓	7009	2990	2371	768

8	8	90	✓	✓	✓	✓	7009	2998	2371	768
10	3	75	✓	✓	✓	✓	3700	1986	1640	618
10	3	80	✓	✓	✓	✓	3700	2007	1640	618
10	3	85	✓	✓	✓	✓	3700	2022	1640	618
10	3	90	✓	✓	✓	✓	3700	2040	1640	618
10	4	75	✓	✓	✓	✓	4148	2180	1640	618
10	4	80	✓	✓	✓	✓	4148	2200	1640	618
10	4	85	✓	✓	✓	✓	4148	2216	1640	618
10	4	90	✓	✓	✓	✓	4148	2240	1640	618
10	5	75	✓	✓	✓	✓	4554	2365	1640	618
10	5	80	✓	✓	✓	✓	4554	2390	1640	618
10	5	85	✓	✓	✓	✓	4554	2415	1640	618
10	5	90	✓	✓	✓	✓	4554	2435	1640	618
10	6	75	✓	✓	✓	✓	4966	2496	1640	618
10	6	80	✓	✓	✓	✓	4966	2526	1640	618
10	6	85	✓	✓	✓	✓	4966	2550	1640	618
10	6	90	✓	✓	✓	✓	4966	2580	1640	618
10	7	75	✓	✓	✓	✓	5364	2642	1640	618
10	7	80	✓	✓	✓	✓	5364	2677	1640	618
10	7	85	✓	✓	✓	✓	5364	2712	1640	618
10	7	90	✓	✓	✓	✓	5364	2726	1640	618
10	8	75	✓	✓	✓	✓	5668	2828	1640	618
10	8	80	✓	✓	✓	✓	5668	2844	1640	618
10	8	85	✓	✓	✓	✓	5668	2876	1640	618
10	8	90	✓	✓	✓	✓	5668	2900	1640	618

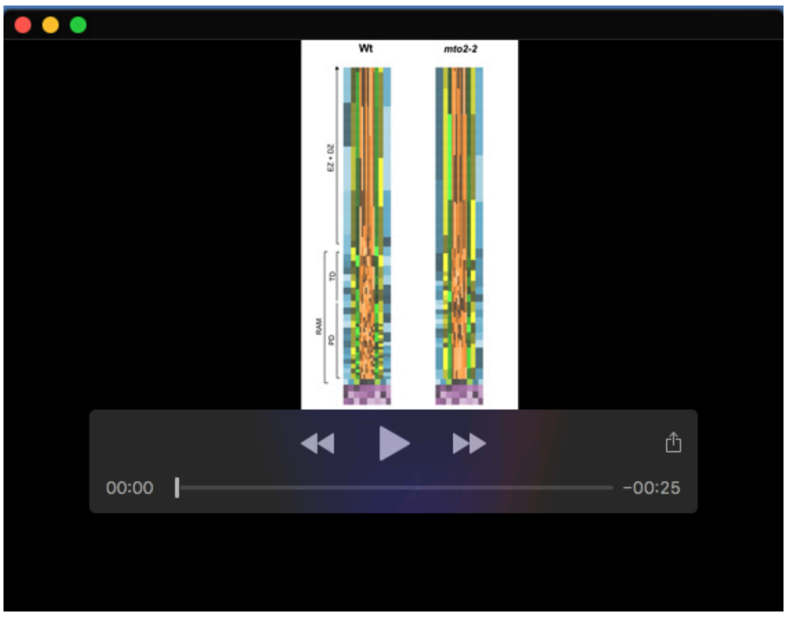

Movie 1. *In silico* simulations of Thr-dependent proliferation dynamics in the RAM of Wt and *mto2-2* roots; related to Fig. 2. Two simulations are shown where the video panels show the cell proliferation dynamics for Wt (left) and *mto2-2* (right) roots. Dynamics are shown from the initial condition where the RAM of Wt and *mto2-2* are identical, until the moment when all the cells of the RAM of the *mto2-2* mutant do not divide anymore and the RAM becomes exhausted (consumed). At this point, cell divisions continue in the Wt *in silico* root. To appreciate cell length distributions along the *in silico* root, each subsequent cell in a cell file is depicted with a different tonality.
