# Supplementary material for: A mutation in THREONINE SYNTHASE 1 uncouples proliferation and transition domains of the root apical meristem: experimental evidence and in silico proposed mechanism
Source: Development. 2022 Nov 9;149(21):dev200899. doi: 10.1242/dev.200899 (PMC9796171; doi:10.1242/dev.200899)
Supplement: Supplementary file 1 [file develop-149-200899_review_history.pdf]

A mutation in THREONINE SYNTHASE 1 uncouples proliferation and transition domains of the root apical meristem: experimental evidence and *in silico* proposed mechanism

Monica L. García-Gómez, Blanca J. Reyes-Hernández, Debee P. Sahoo, Selene Napsucialy-Mendivil, Aranza X. Quintana-Armas, José A. Pedroza-García, Svetlana Shishkova, Héctor H. Torres-Martínez, Mario A. Pacheco-Escobedo and Joseph G. Dubrovsky
DOI: 10.1242/dev.200899

Editor: Yka Helariutta

Review timeline
Original submission: 28 April 2022
Editorial decision: 7 June 2022
First revision received: 8 September 2022
Accepted: 10 October 2022

Original submission

First decision letter

MS ID#: DEVELOP/2022/200899

MS TITLE: A mutation in THREONINE SYNTHASE 1 uncouples proliferation and transition domains of the root apical meristem: experimental evidence and in silico proposed mechanism

AUTHORS: Monica L. García-Gómez, Blanca Jazmín Reyes-Hernández, Debee Prasad Sahoo, Selene Napsucialy-Mendivil, Aranza Xhaly Quintana-Armas, José Antonio Pedroza-García, Svetlana Shishkova, Héctor Hugo Torres-Martínez, Mario A. Pacheco-Escobedo, and Joseph G. Dubrovsky

I have now received all the referees' reports on the above manuscript, and have reached a decision. The referees' comments are appended below, or you can access them online: please go to BenchPress and click on the 'Manuscripts with Decisions' queue in the Author Area.

As you will see, the referees express considerable interest in your work, but have some significant criticisms and recommend a substantial revision of your manuscript before we can consider publication. If you are able to revise the manuscript along the lines suggested, which may involve further experiments, I will be happy receive a revised version of the manuscript. Your revised paper will be re-reviewed by one or more of the original referees, and acceptance of your manuscript will depend on your addressing satisfactorily the reviewers' major concerns. Please also note that Development will normally permit only one round of major revision. If it would be helpful, you are welcome to contact us to discuss your revision in greater detail. Please send us a point-by-point response indicating your plans for addressing the referee’s comments, and we will look over this and provide further guidance.

Please attend to all of the reviewers' comments and ensure that you clearly highlight all changes made in the revised manuscript. Please avoid using 'Tracked changes' in Word files as these are lost in PDF conversion. I should be grateful if you would also provide a point-by-point response detailing how you have dealt with the points raised by the reviewers in the 'Response to Reviewers' box. If you do not agree with any of their criticisms or suggestions please explain clearly why this is so.

© 2022. Published by The Company of Biologists under the terms of the Creative Commons Attribution License (https://creativecommons.org/licenses/by/4.0/).

Reviewer 1

Advance summary and potential significance to field

In this manuscript, the authors reported the role of threonine metabolism in root meristem maintenance. Through detailed phenotypical quantification, the authors proposed that the root meristem of mto2 mutant is composed of only a single domain that phenotypically resembles the transition domain (TD).
Consistently, the CCS52A1, a molecular marker of TD, was expressed in all RM cells in mto2 mutant. Furthermore, the well-known RAM maintenance marker, such as WOX5 and PLTs, were normally expressed in mto2, suggesting that threonine acts RAM maintenance independent of those regulatory pathways. Since there is no method to directly measure the spatial distribution pattern of threonine, the authors conducted mathematical modeling, which predicts the highest threonine synthesis and catabolism in the SNC of the root meristem. The authors proposed that threonine metabolism in mto2 mutant is most affected in this SNC region suggesting that a deficiency of threonine in the SCN is the underlying cause of the mto2 mutant phenotype. In this manuscript, the authors conducted a unique approach to understanding the link between root meristem maintenance and threonine metabolism by combining molecular genetics and mathematical modeling which provides new insight into root organogenesis. However, I found several issues that needed to be resolved before publication.

Comments for the author

Major points;
- One major concern in this study is the difference in the prediction of threonine distribution between two theoretical models presented in Fig. 2 and 7.
In the model in Fig. 2, the authors claimed that the highest threonine level is observed in the SCN in the wild-type meristem and decreased toward the proximal side of the root meristem, which is consistent with the prediction shown in Fig. 7a. On the other hand, the extended model in Fig. 7B in the wild type showed threonine concentration becomes higher toward the proximal side of the root meristem, which later damped temporally and further increased to the differentiation zone (to the region indicated as “1000 a.u.”). In Fig. 2, the authors established the model connecting the threonine level and cell proliferation, where cell division is allowed as long as the free threonine level is above a threshold. With this model, they simulated the cell behavior of mto2 mutant, predicting the TD-like cell behavior of RAM cells in mto2. If the threonine distribution is formed as predicted in Fig. 7b, I would imagine that proliferation pattern and growth must be differently predicted; however, there is no theoretical modeling simulating the cell behavior with the threonine distribution predicted in Fig 7b. Since there are non-negligible differences in the threonine distribution in their models (Fig. 2, 7a compared to Fig 7b), the authors should apply the cell proliferation modeling to the threonine distribution pattern predicted in Fig. 7b. Otherwise, it is difficult for readers to understand how the authors propose the relationship between compartmentalization of threonine metabolic activity and root cell proliferation.
- Another major concern in this study is the biological validity of their theoretical models. It seems that the authors used the data of the transcriptional profiling published in Wendrich et al. to incorporate the expression pattern of each gene into their modeling. If I understand correctly the data set in Wendrich et al. only includes the cell in the root meristem
(indicated as plots in Fig. 7). I wonder how the authors set the expression of those elements in the other domain, such as the differentiation zone. Since the prediction of their model largely depends on the expression pattern of enzymes including MTO2, TSY2, THAs, and OMR1, the authors have to evaluate their assumption of gene expression patterns by biological experiments, such as qRT-PCR or reporter lines. For example, the authors wrote that “MTO2 is highly expressed in the RAM, and its expression gradually decreases toward the elongation and differentiation zones. This coincides with the proMTO2:MTO2-GFP expression pattern (Reyes-Hernández et al., 2019), indicating that a protein graded distribution is similar to the MTO2 transcriptional gradient (line666-669)”; however, I could not see such expression gradient of MTO2-GFP in the root meristem from the image shown in Reyes-Hernández et al., 2019.
Additionally, I could not understand how the authors assumed the expression of TSY2, especially in the differentiation zone, which showed the drastic increase in its expression at the domain far from meristem (800 a.u. in Fig7A). Again the predictions from their model largely depend on the

© 2022. Published by The Company of Biologists under the terms of the Creative Commons Attribution License (https://creativecommons.org/licenses/by/4.0/).

expression pattern of enzymes (this idea is consistent with the fact that other parameters, such as each Km, did not affect the pattern of threonine distribution in their model),
the authors need to provide the biological evidence of the spatial expression pattern of each enzyme in the root.
- Through their theoretical approaches, the authors claimed that a deficiency of threonine in the SCN is the underlying cause of the mto2-2 phenotype (line417-418). This is an essential point in this manuscript, and it could be further evaluated by molecular genetics. For example, the author could express MTO2 under SCN promoter, such as PLT promoter, thereby examining how the phenotype of mto2 could be rescued. Since the biological evaluation of their mathematical model is limited in the current manuscript, an additional experiment is required to support the authors' claim.
Minor point In Fig. 2D, the difference in the color of each cell type is not mentioned. Is this indicate the difference in threonine level?

Reviewer 2

Advance summary and potential significance to field

Root growth depends on the production of new cells and their differentiation, including enlargement, into numerous cell types. The role of stem cells is crucial, but also the proliferation of their progeny. In this study authors continue studying an Arabidopsis mutant severely affected in the proliferation domain of the root meristem. Interestingly, the mutant is defective in an enzyme of the threonine metabolism. Although the mutant has been already published earlier, the current study still holds sufficient novelty as it aimed at identifying the mechanism involved using a combination of wet and computer simulation strategies. It also is relevant because the study goes beyond the more heavily studied gene networks controlling root growth.

Comments for the author

A general comment that applies to several sections and figures: experiments should be made at comparable times after germination. In fact, it would be better to have a detailed analysis at several times after germination in all cases, and not only at a given time, sometimes early, sometimes much later. Analysis of reporter expression in the mto2-2 background (Fig. 4, 5, 6) should be analyzed at the cellular level in a time course from 1 dag to complete meristem exhaustion.
Specific comments on weak points that need to be addressed in order to improve and/or clarify the results and conclusions of this work.
1. Line 129. The use of "10 distal cells" in the measurements needs to be justified properly.
2. Line 139. Since the concepts of PD and TD are a matter of debate in the field, it would be good to provide more details (not only a reference) about how these domains and their boundary is defined experimentally.
3. Line 146. Do other known mutants with exhausted root meristem behave as the mto2-2 mutant? Is this finding general for all (or many) mutations leading to meristem exhaustion?
4. Line 156. Fig 2A. Images of the two markers mentioned should be included to clarify the presence of opposing expression gradients.
5. Line 250 and 310. The case of CCS52A1 expression is very relevant. It seems, although not fully proven that expression of this marker (reported by the authors to define the TD), is activated at positions very distal ion the meristem. Does this mean that CCS52A1 is expressed already in one of the daughter cells after the asymmetric division of a stem cell? Does this daughter cell enter endoreduplication immediately after division or does it divide once? Twice? Or never? What is the fate of the daughter cells after division of one stem cell?
Does it express the endoreduplication marker right after division? Does it express known cell identity genes after division of a stem cell? A detailed analysis at the individual cell level is necessary to fully understand the fate of daughter cells in the mutant.
6. What is the pattern of CYCB1;1 expression in the mto2-2 mutant at different times after germination?
And the pattern of EdU labelling? The combination of these would be very enlightening regarding the fate of daughter cells after division of stem cells to ascertain whether they are actually in a functional TD, as claimed by the authors.

© 2022. Published by The Company of Biologists under the terms of the Creative Commons Attribution License (https://creativecommons.org/licenses/by/4.0/).

Reviewer 3

Advance summary and potential significance to field

Manuscript by García-Gómez et al., is an interesting combination of experimental and modeling work that attempt to reveal mechanism that separates transition zone from active root meristem that require Thr metabolism. The idea seems new and hypothesis is sound. I would like to comment on modeling part as this is my primary field of expertise.

Comments for the author

I have several questions regarding presentation and definition of the models that I would like authors to address:
1) Model assumes fixed gene expression patterns to predict Thr distribution in the root. How robust results are if the enzyme expression changes over time?
Could that be tested in the model?
2) It is not clear how cell division and elongation are simulated and what are underlying assumptions. For instance, model assumes infinite proliferation capacity that depends only on Thr. This clearly not the only regulator and no discussion is provided how other known regulators such as PLETHORA would contribute to these predictions. Other, how is maximal cell length selected? I must admit had a hard time of finding this information in SI. Again, how robust are results to changes of model parameters that define cell growth?
3) Video S1 is missing I would like to see the actual dynamics of the model
4) Fig. 2D is very cryptic. What those different intensities stand for?
Frankly, I do not know what should I understand form this figure.
5) Generally, model description should be improved and made clear for not modelers and analysis of parameter sensitivity and model robustness seems missing.

First revision

Author response to reviewers' comments

Reviewer 1 Advance Summary and Potential Significance to Field:

In this manuscript, the authors reported the role of threonine metabolism in root meristem maintenance. Through detailed phenotypical quantification, the authors proposed that the root meristem of mto2 mutant is composed of only a single domain that phenotypically resembles the transition domain (TD). Consistently, the CCS52A1, a molecular marker of TD, was expressed in all RAM cells in mto2 mutant. Furthermore, the well-known RAM maintenance marker, such as WOX5 and PLTs, were normally expressed in mto2, suggesting that threonine acts RAM maintenance independent of those regulatory pathways. Since there is no method to directly measure the spatial distribution pattern of threonine, the authors conducted mathematical modeling, which predicts the highest threonine synthesis and catabolism in the SNC of the root meristem. The authors proposed that threonine metabolism in mto2 mutant is most affected in this SNC region, suggesting that a deficiency of threonine in the SCN is the underlying cause of the mto2 mutant phenotype. In this manuscript, the authors conducted a unique approach to understanding the link between root meristem maintenance and threonine metabolism by combining molecular genetics and mathematical modeling, which provides new insight into root organogenesis. However, I found several issues that needed to be resolved before publication.

Reviewer 1 Comments for the Author:
Major points;

-One major concern in this study is the difference in the prediction of threonine distribution between two theoretical models presented in Fig. 2 and 7. In the model in Fig. 2, the authors claimed that the highest threonine level is observed in the SCN in the wild-type meristem and

© 2022. Published by The Company of Biologists under the terms of the Creative Commons Attribution License (https://creativecommons.org/licenses/by/4.0/).

decreased toward the proximal side of the root meristem, which is consistent with the prediction shown in Fig. 7a. On the other hand, the extended model in Fig. 7B in the wild type showed threonine concentration becomes higher toward the proximal side of the root meristem, which later damped temporally and further increased to the differentiation zone (to the region indicated as "1000 a.u."). In Fig. 2, the authors established the model connecting the threonine level and cell proliferation, where cell division is allowed as long as the free threonine level is above a threshold. With this model, they simulated the cell behavior of mto2 mutant, predicting the TD-like cell behavior of RAM cells in mto2. If the threonine distribution is formed as predicted in Fig. 7b, I would imagine that proliferation pattern and growth must be differently predicted; however, there is no theoretical modeling simulating the cell behavior with the threonine distribution predicted in Fig 7b. Since there are non-negligible differences in the threonine distribution in their models (Fig. 2, 7a compared to Fig 7b), the authors should apply the cell proliferation modeling to the threonine distribution pattern predicted in Fig. 7b. Otherwise, it is difficult for readers to understand how the authors propose the relationship between compartmentalization of threonine metabolic activity and root cell proliferation.

R= We would like to thank Reviewer 1 for all the positive comments about the originality of our approach and the importance of the Thr pathway we report in our manuscript and his/her recommendations on the improvement of our work. As mentioned by the reviewer, the free threonine (Thr) distributions predicted by the two models are quite different. Yet, both are consistent with our proposal that Thr synthesis is the highest at the root stem cell niche (SCN), with model B exploring additional layers of Thr metabolism in the root apical meristem (RAM). Indeed, considering the role of Thr catabolism, the model predicts very low levels of Thr in the root SCN suggesting that the SCN in addition to being a site of high Thr synthesis, it is also a site of high Thr catabolism. As pointed out by the reviewer, we did not explore the developmental consequences of Thr catabolism in the *in silico* growing root in our original submission. We agree with this comment and, therefore, performed simulations to address the output of Thr catabolism on proliferation activity in the PD of the RAM (Figure S12). This updated model recovered that Wt roots grow indeterminately, whereas the *mto2-2* roots do not, a result equivalent to the one with model A. In model B, the Wt simulation results in optimal levels of Thr catabolic products in the proliferation domain (PD) of the RAM (capable of supporting active cell proliferation), while in the *mto2-2* simulations there is a deficiency of these metabolites as a consequence of the loss of MTO2 in the root apex. Ultimately, both models are compatible in the sense that they show that Thr (either as a result of only synthesis or synthesis and catabolism) is a fundamental metabolite to support proliferation in the RAM. We included the results of this simulation in the extended supplementary information making reference in the main text where we discuss the two alternative Thr metabolism models. The respective changes in the text appear on lines 434-439 and a new figure appears as Fig. S12.

-Another major concern in this study is the biological validity of their theoretical models. It seems that the authors used the data of the transcriptional profiling published in Wendrich et al. to incorporate the expression pattern of each gene into their modeling. If I understand correctly, the data set in Wendrich et al. only includes the cell in the root meristem (indicated as plots in Fig. 7). I wonder how the authors set the expression of those elements in the other domain, such as the differentiation zone

R= The data used for the model include not only the study of Wendrich et al., (2017) covering three segments of the RAM, but also three transcriptomic studies covering a larger segment of the root (Brady et al., 2007; Denyer et al., 2019; Zhang et al., 2019), two of them are recent single-cell transcriptomic datasets that are publicly available, and this information was exploited to derive the expression patterns of the enzymes. It is from these data that we noticed a high expression level of *TSY2* in the differentiation zone in two independent studies (Brady et al., 2007; Zhang et al., 2019); this sudden increase in *TSY2* expression was not found for the other enzymes considered in our model. Based on these data we defined the expression of the enzymes in the differentiation zone: high *TSY2* expression and minimal expression levels for the rest of the enzymes. Importantly, the expression of *TSY2* is not necessary to explain the determinate growth phenotype of *mto2-2* roots (Fig. S3), and thus the main conclusions of our study. Still, its activity in the DZ is necessary to explain the higher free Thr content in *mto2-2* roots (confirming experimental data published before), while in the RAM it explains the few cell divisions that still take place in the *mto2-2* mutant (Fig. S3), supporting the addition of TSY2 in the model. This example nicely illustrates how

© 2022. Published by The Company of Biologists under the terms of the Creative Commons Attribution License (https://creativecommons.org/licenses/by/4.0/).

mathematical modelling can yield new conclusions given existent data. This is important also as an example of how existing expression datasets can be exploited as a resource to learn about the role of genes (i.e. *TSY2*) in root development. We now mention all this information in the new version of the text (lines 213-214).

Since the prediction of their model largely depends on the expression pattern of enzymes, including MTO2, TSY2, THAs, and OMR1, the authors have to evaluate their assumption of gene expression patterns by biological experiments, such as qRT- PCR or reporter lines. For example, the authors wrote that "MTO2 is highly expressed in the RAM, and its expression gradually decreases toward the elongation and differentiation zones. This coincides with the proMTO2:MTO2-GFP expression pattern (Reyes-Hernández et al., 2019), indicating that a protein graded distribution is similar to the MTO2 transcriptional gradient (line666-669)"; however, I could not see such expression gradient of MTO2-GFP in the root meristem from the image shown in Reyes-Hernández et al., 2019 Additionally, I could not understand how the authors assumed the expression of TSY2, especially in the differentiation zone, which showed the drastic increase in its expression at the domain far from meristem (800 a.u. in Fig7A). Again, the predictions from their model largely depend on the expression pattern of enzymes (this idea is consistent with the fact that other parameters, such as each Km, did not affect the pattern of threonine distribution in their model), the authors need to provide the biological evidence of the spatial expression pattern of each enzyme in the root.

R= The predictions of the model do depend on the expression pattern of the enzymes, particularly of *MTO2* (see simulation with no *TSY2* expression, Fig. S3). Here we describe the different sources of experimental data that support the expression patterns of the enzymes considered in the model. We previously showed (but did not measure) that *MTO2* is highly expressed in the root SCN while its expression decreases gradually up to the elongating cells (Reyes-Hernández et al., 2019). Here, to address this critique and validate that the *MTO2* expression decreases gradually towards the differentiation zone, we performed a quantitative analysis of the GFP intensity using the *pMTO2:MTO2-GFP* reporter created in our laboratory. We quantified the relative GFP signal in cells at different distances from the QC and the data are included in Fig. S2. We also performed the q-RT-PCR analysis of *MTO2* and *TSY2* expression, and the data are included in the same figure (Fig. S2). In the revised manuscript this is mentioned now on lines 163-172. In regard to the other enzymes, we used three different sources of transcriptomic data that show: a decreasing gradient pattern for *OMR1*, *THA1,* and *THA2* (Wendrich et al., 2019; Denyer et al., 2019; Zhang et al., 2019) and a low expression and high expression pattern for *TSY2* in the RAM and differentiated cells, respectively (Brady et al., 2007; Wendrich et al., 2019; Zhang et al., 2019). We believe it is important to use existing datasets as a resource to advance the understanding of root development, and in this case the expression patterns included in the model are based on more than one dataset providing confidence in them. Also it is important to mention that the main conclusions of our model regarding Thr as a metabolite necessary to maintain indeterminate root growth can be explained solely by the expression of *MTO2*, of which we clearly show the existence of the gradient along the root. Nevertheless, adding *TSY2* expression does explain the higher free Thr content in *mto2-2* roots (Fig. S3), while adding *OMR1, THA1* and *THA2* allowed us to also reveal the root stem cell niche as a region of high Thr catabolism (Fig. 7B). These additional layers of information are thus necessary to further understand the phenotype of *mto2-2* beyond this single gene.

-Through their theoretical approaches, the authors claimed that a deficiency of threonine in the SCN is the underlying cause of the mto2-2 phenotype (line417-418). This is an essential point in this manuscript, and it could be further evaluated by molecular genetics. For example, the author could express MTO2 under SCN promoter, such as PLT promoter, thereby examining how the phenotype of mto2 could be rescued. Since the biological evaluation of their mathematical model is limited in the current manuscript, an additional experiment is required to support the authors' claim.

R= We agree that the experiment proposed by the reviewer would be an excellent way to unequivocally validate the predictions derived from our computational model in regard to the SCN being the most affected region. Indeed, this is something we are currently exploring in the laboratory to further understand the role of MTO2 in the root SCN. Nevertheless, in the present manuscript, our aim was to analyze the relationship of free Thr with the proliferation activity and growth pattern and to show that the transition domain of the RAM can be unlinked from the

© 2022. Published by The Company of Biologists under the terms of the Creative Commons Attribution License (https://creativecommons.org/licenses/by/4.0/).

proliferation domain. Here our main proposal is that Thr metabolism is compartmentalized in the RAM. With our experimental and computational approaches we provide clear evidence that the *MTO2* expression gradient is sufficient to reproduce the *mto2-2* phenotype *in silico* (i.e. determinate growth, cell length profile similarity to the RAM PD in Wt roots), while the model simulations provide novel predictions of the importance of *TSY2* in the differentiation zone to explain the high free Thr content in the mutant root. We consider that these predictions support our hypothesis that free Thr as a critical factor coupling the PD and the TD in the RAM, and while the model also predicts its necessity for the SCN, the proposed experiment are out of the goals of the presented work. Importantly, our computational modelling approach confirmed conclusions derived from cell length profile and MSC analysis pointing out that the RAM can exist without a PD, thereby providing a new understanding of the RAM organization and maintenance. We mention this on lines 512-517 of the manuscript.

Minor point
In Fig. 2D, the difference in the color of each cell type is not mentioned. Is this indicate the difference in threonine level?

R= The different colour shades indicate individual cells, with the each representing different cell types (i.e., epidermis - blue, cortex - yellow, endodermis - green, pro-vascular tissues - orange, QC - dark gray, and columella - purple). We are sorry for the omission of a clear explanation. Now we added to the legend of Figure 2 the following text: “To appreciate cell length distributions along the *in silico* root, each subsequent cell in a cell file is depicted with a different tonality.” We also added to this legend the following text: “Note that indications of the PD and TD of the RAM refer only to Wt and the *mto2-2* at time $t_{1;}$ in the *mto2-2* mutant at time t_2, cell length profile reproduces absence of PD cells similar to real data shown in Fig 1B”.

Reviewer 2 Advance Summary and Potential Significance to Field:
Root growth depends on the production of new cells and their differentiation, including enlargement, into numerous cell types. The role of stem cells is crucial, but also the proliferation of their progeny. In this study, authors continue studying an Arabidopsis mutant severely affected in the proliferation domain of the root meristem. Interestingly, the mutant is defective in an enzyme of the threonine metabolism. Although the mutant has been already published earlier, the current study still holds sufficient novelty as it aimed at identifying the mechanism involved using a combination of wet and computer simulation strategies. It also is relevant because the study goes beyond the more heavily studied gene networks controlling root growth.

Reviewer 2 Comments for the Author:

A general comment that applies to several sections and figures: experiments should be made at comparable times after germination. In fact, it would be better to have a detailed analysis at several times after germination in all cases, and not only at a given time, sometimes early, sometimes much later. Analysis of reporter expression in the mto2-2 background (Fig. 4, 5, 6) should be analyzed at the cellular level in a time course from 1 dag to complete meristem exhaustion.

R= We thank the reviewer for this comment. Indeed, a reader can have an impression that the experimental design is not well planned. However, we characterized before that the *mko1/mto2-2* mutant shows much heterogeneity in the beginning and advancement of the RAM exhaustion (Hernández-Barrera *et al.*, 2011; Reyes-Hernandez et al., 2019). For this reason, plants shown in Figures 5 and 6 range from 7 to 11 dag in the *mto2-2* mutant. We show either the root tip when the RAM is present (left panels of Figure 5), or when the RAM is completely or almost completely exhausted (right panels of the Figure 5). To make it clearer we added the respective information in the legend to Figures 5 and 6 of the revised version of our manuscript. In Figure 4, we show the expression of *pCCS52A1::CCS52A1-GFP* at a stage when the RAM is present in the mutant; so 3 dag was selected for illustration. We mention that the arrow indicates a boundary between the RAM and the elongation zone, and thus hope it is clear that the RAM is present at this stage. To be explicit we also added the information that the process of the RAM exhaustion in *mto2-2* is heterogeneous (lines 276-277).

© 2022. Published by The Company of Biologists under the terms of the Creative Commons Attribution License (https://creativecommons.org/licenses/by/4.0/).

Specific comments on weak points that need to be addressed in order to improve and/or clarify the results and conclusions of this work.
1.Line 129. The use of "10 distal cells" in the measurements needs to be justified properly.

R= We explain this detail on lines 134-136 with the following text: We chose to measure 10 distal cells because our analysis showed this number corresponds to the RAM length in the *mto2-2* mutant before the RAM exhaustion (see below and Table S1).

2.Line 139. Since the concepts of PD and TD are a matter of debate in the field, it would be good to provide more details (not only a reference) about how these domains and their boundary is defined experimentally.

R= Thank you for this comment. We added the respective text on lines 471-477.

3.Line 146. Do other known mutants with exhausted root meristem behave as the mto2-2 mutant? Is this finding general for all (or many) mutations leading to meristem exhaustion?

R= Among the mutants known to cause determinate root growth in Arabidopsis (i.e. *short root*, *scr*, *plt1 plt2* and others) the alterations in the meristematic domains have not been characterized to date with the integrative approach we used in this manuscript to define the domains of the RAM (i.e. cell length profile analysis, mathematical modelling, and cell cycle molecular markers). Arbitrarily, the mentioned mutants may have their RAM also represented mainly by the TD, but further research is required. As we already have passed the word limit in our manuscript, we decided not to mention about this here. However,we added a short note in the Discussion section that address the raised question on lines 530-532.

4.Line 156. Fig 2A. Images of the two markers mentioned should be included to clarify the presence of opposing expression gradients.

R= We have now included the results of a quantitative analysis of the GFP distribution in the *pMTO2:MTO2-GFP* reporter line and qRT-PCR analysis of *MTO2* and *TSY2* expression (Figure S2).

5.Line 250 and 310. The case of CCS52A1 expression is very relevant. It seems, although not fully proven, that expression of this marker (reported by the authors to define the TD), is activated at positions very distal in the meristem. Does this mean that CCS52A1 is expressed already in one of the daughter cells after the asymmetric division of a stem cell? Does this daughter cell enter endoreduplication immediately after division or does it divide once? Twice? Or never? What is the fate of the daughter cells after division of one stem cell? Does it express the endoreduplication marker right after division? Does it express known cell identity genes after division of a stem cell? A detailed analysis at the individual cell level is necessary to fully understand the fate of daughter cells in the mutant.

R= We thank the reviewer for this important comment. We added respective text on lines 506-511: "As *pCCS52A1::CCS52A1-GFP* in the *mto2-2* mutant is expressed in the ground tissue and columella stem cells (Fig. 4), the question arises whether these stem cells pass through at least one cell division cycle before entering the endoreduplication. Among all *mto2-2* roots imaged with various approaches described (n > 210), only one root was found where stem cells divided (Fig. S7), suggesting that proliferation of the QC cells (Fig. 6B) is accompanied by an arrest in stem cell activity".

6.What is the pattern of CYCB1;1 expression in the mto2-2 mutant at different times after germination? And the pattern of EdU labelling? The combination of these would be very enlightening regarding the fate of daughter cells after division of stem cells to ascertain whether they are actually in a functional TD, as claimed by the authors.

R= We previously reported the activity of *CYCB1;1-GFP* in the *mko1/mto2-2* background and showed low but present proliferation activity (Hernández-Barrera et al., 2011). We agree that the proposed experiment would be useful to understand relationship between cell cycle and endoreduplication in the mutant. Unfortunately, the *mko1 CYCB1;1-GFP* created in my laboratory 12 years ago behaved atypically, and we did not have now sufficient time to obtain this line again as the second

© 2022. Published by The Company of Biologists under the terms of the Creative Commons Attribution License (https://creativecommons.org/licenses/by/4.0/).

generation of the cross is required. As outlined in the response to point 4, as an alternative we performed EdU experiments (Fig. S7), and also a time-lapse analysis of AtPCNA (Fig. S6). This new material is included on lines 269-281.

Reviewer 3 Advance Summary and Potential Significance to Field:

Manuscript by García-Gómez et al., is an interesting combination of experimental and modeling work that attempt to reveal mechanism that separates transition zone from active root meristem that require Thr metabolism. The idea seems new and hypothesis is sound. I would like to comment on modeling part as this is my primary field of expertise.

Reviewer 3 Comments for the Author:

I have several questions regarding presentation and definition of the models that I would like authors to address:

1) Model assumes fixed gene expression patterns to predict Thr distribution in the root. How robust results are if the enzyme expression changes over time? Could that be tested in the model?

R= Yes, this can be tested with the model. In fact, that is an advantage of using a computational modelling approach that can allow us to explore conditions that have not been described experimentally (most studies focus on young seedlings), and then to generate novel predictions. This is relevant because most studies focus on a fixed stage in root development (usually 3 or 5 dag) and how the molecular mechanisms behave through time remains not completely understood. Thus, we used our simulation platform to test what would be the consequences of decreasing and increasing the expression level of enzymes involved in Thr metabolism important for root growth. We decided to simulate gradual increases / decreases of gene expression, as we do not have evidence of the particular tendency of these changes in gene expression through time for the enzymes considered in the model. We found that the results of our model's are robust to the changes in gene expression we implemented (Figure S13). Importantly, it has been shown that as roots age, the expression of several stem cell markers drastically decrease in the QC cells (*WOX5*: Timilsina et al., 2019; *QC184*: Wein et al., 2020), with implications in long-term root growth. Therefore, of these two hypothetical scenarios, decreasing gene expression could provide interesting predictions with functional implications, namely to how roots behave as they age. Importantly, our aim in this manuscript was to propose the mechanism of RAM exhaustion in the *mto2-2* mutant and the Thr-dependent mechanisms for meristem maintenance in Wt roots. Thus, for the default simulations presented here we use publicly available data of studies young seedlings, as it is in this time frame that the meristem is consumed in *mto2-2* roots. As the RAM in Wt roots is not consumed, we consider that the expression patterns do not change significantly in our default simulations (Fig. 2, 7, Fig. S3, S12).

We performed the analysis of the robustness of our model, which is included in the Materials and Methods, lines 737-759.

2) It is not clear how cell division and elongation are simulated and what are underlying assumptions. For instance, model assumes infinite proliferation capacity that depends only on Thr. This clearly not the only regulator and no discussion is provided how other known regulators such as PLETHORA would contribute to these predictions. Other, how is maximal cell length selected? I must admit had a hard time of finding this information in SI. Again, how robust are results to changes of model parameters that define cell growth?

R= We addressed the raised question in the Materials and Methods section, on lines 714-719.

Regarding other regulators of RAM maintenance, particularly the PLETHORA transcription factors, here we show that the expression of *PLT1* in the SCN is not compromised in the *mto2-2* mutant (Figure 6A). This is indicative that the free Thr pathway we describe acts independently of the PLT-pathway. For this reason, we did not incorporate the PLT dynamics in our model simulations. Still we agree that the PLT pathway is very important for root continuous growth, and as pointed out by

© 2022. Published by The Company of Biologists under the terms of the Creative Commons Attribution License (https://creativecommons.org/licenses/by/4.0/).

the reviewer, it is of interest to understand how these pathways are integrated in RAM regulation. We mention this in the Discussion section (lines 523-532).

Regarding the parameters related to cell growth dynamics of the *in silico* root, we performed a parameter analysis and summarized the results in Table S5, and included the respective text in the "Materials and Methods" section that appears on lines 737-749.

3) Video S1 is missing I would like to see the actual dynamics of the model -

R= Video S1 is part of the supplementary information that was included in our original submission.

4) Fig. 2D is very cryptic. What those different intensities stand for? Frankly, I do not know that should I understand form this figure.

R= We apologize for the confusion. Indeed, this point was also brought up by reviewer 1. The simulation platform we developed does not define cell walls explicitly. This is now explained in the figure legend. The reason we include panel D in Figure 2 is to show where the cell length distributions analysed.

5) Generally, model description should be improved and made clear for not modelers and analysis of parameter sensitivity and model robustness seems missing.

R= As explained to in the comment regarding the robustness of the model results, we performed an additional analysis and added the respective text in the Materials and Methods section (lines 737-759).

Second decision letter

MS ID#: DEVELOP/2022/200899

MS TITLE: A mutation in THREONINE SYNTHASE 1 uncouples proliferation and transition domains of the root apical meristem: experimental evidence and in silico proposed mechanism

AUTHORS: Monica L. García-Gómez, Blanca Jazmín Reyes-Hernández, Debee Prasad Sahoo, Selene Napsucialy-Mendivil, Aranza Xhaly Quintana-Armas, José Antonio Pedroza-García, Svetlana Shishkova, Héctor Hugo Torres-Martínez, Mario A. Pacheco-Escobedo, and Joseph G. Dubrovsky
ARTICLE TYPE: Research Article

I am happy to tell you that your manuscript has been accepted for publication in Development, pending our standard ethics checks.

Reviewer 1

Advance summary and potential significance to field

First, I would like to appreciate the author answering my previous concerns by adding new results. Especially, I appreciate that the authors added a new theoretical growth model with incorporating the Thr metabolism and synthesis which is consistent with the authors' claim in the original manuscript. In addition to that, the authors have conducted several additional biological experiments. One critical data showing the expression gradient of MTO2 in vivo has been quantitated in the current manuscript, which further supports their claim. Though I have suggested an experiment that evaluates the functional significance of MTO2 expression in SCN by using PLT promoter, I fully agree with the authors' opinion that my proposal is out of the goals of the presented work.

© 2022. Published by The Company of Biologists under the terms of the Creative Commons Attribution License (https://creativecommons.org/licenses/by/4.0/).

Comments for the author

I think the current manuscript provides an important insight into root development, which is suitable for publishing in Development.

Reviewer 2

Advance summary and potential significance to field

This manuscript shows the role of the gene under study in maintaining a proliferation domain within the root apical meristem. This is a valuable advance to the field, provided that authors fully address my comments
(see below).

Comments for the author

I appreciate that authors have tried to address my comments. They have argued in a convincing manner in most cases. There is, however, one point that it is not yet fully and clearly addressed. This refers to Point 6 I my original review, related to provide a more detailed and consistent analysis of division and CCS52 expression over the time after germination. Authors refer to a previous publication where CYCB1;1 expression was analyzed at 1 and 6 days after germination. To have a full support to their claims, it was asked to visualize the expression pattern at various times between 1 and 6 days. They argue that this line has been lost, that it is no longer available and that it will take too long to generate a new one. While this is probably not the best answer, I accept it because there is an alternative. In order to demonstrate that the PD is absent in the mutant an experiment can be done by analyzing the CCS52 expression (a marker of TD), on a daily basis, after germination, at least 1, 3 and 6 days would serve to support their claim of a loss of PD in the mutant. This will also serve to confirm in an independent manner the data provided in Table S1, where it is shown that the mutant has less than 1 cell in this domain, on average.

Reviewer 3

Advance summary and potential significance to field

This manuscript shows an interesting link between activity of cell proliferation
and synthesis of amino acid threonine in root meristem . Bothe experimental and theoretical findings are new and thus expand our understanding the regulation of cell division in the root meristem.

Comments for the author

Authors has satisfactory revised the manuscript and answered most of my concerns/suggestions. I am happy to recommend this paper for publication.

© 2022. Published by The Company of Biologists under the terms of the Creative Commons Attribution License (https://creativecommons.org/licenses/by/4.0/).
